# Two transcription factors, AcREM14 and AcC3H1, enhance the resistance of kiwifruit *Actinidiachinensis* var. *chinensis* to* Pseudomonas syringae* pv. *actinidiae*

**DOI:** 10.1093/hr/uhad242

**Published:** 2023-11-20

**Authors:** Chao Zhao, Wei Liu, Yali Zhang, Yuanzhe Li, Chao Ma, Runze Tian, Rui Li, Mingjun Li, Lili Huang

**Affiliations:** State Key Laboratory of Crop Stress Biology for Arid Areas, College of Plant Protection, Northwest A&F University, Xianyang 712100, China; State Key Laboratory of Crop Stress Biology for Arid Areas, College of Plant Protection, Northwest A&F University, Xianyang 712100, China; State Key Laboratory of Crop Stress Biology for Arid Areas, College of Plant Protection, Northwest A&F University, Xianyang 712100, China; State Key Laboratory of Crop Stress Biology for Arid Areas, College of Plant Protection, Northwest A&F University, Xianyang 712100, China; State Key Laboratory of Crop Stress Biology for Arid Areas, College of Plant Protection, Northwest A&F University, Xianyang 712100, China; State Key Laboratory of Crop Stress Biology for Arid Areas, College of Plant Protection, Northwest A&F University, Xianyang 712100, China; State Key Laboratory of Crop Stress Biology for Arid Areas, College of Plant Protection, Northwest A&F University, Xianyang 712100, China; State Key Laboratory of Crop Stress Biology in Arid Areas, College of Horticulture, Northwest A&F University, Yangling, Shaanxi 712100, China; State Key Laboratory of Crop Stress Biology for Arid Areas, College of Plant Protection, Northwest A&F University, Xianyang 712100, China

## Abstract

Kiwifruit bacterial canker is a global disease caused by *Pseudomonas syringae* pv. *actinidiae* (*Psa*), which poses a major threat to kiwifruit production worldwide. Despite the economic importance of *Actinidia chinensis* var. *chinensis*, only a few resistant varieties have been identified to date. In this study, we screened 44 kiwifruit *F*_1_ hybrid lines derived from a cross between two *A. chinensis* var. *chinensis* lines and identified two offspring with distinct resistance to *Psa*: resistant offspring RH12 and susceptible offspring SH14. To identify traits associated with resistance, we performed a comparative transcriptomic analysis of these two lines. We identified several highly differentially expressed genes (DEGs) associated with flavonoid synthesis, pathogen interactions, and hormone signaling pathways, which play essential roles in disease resistance. Additionally, using weighted gene co-expression network analysis, we identified six core transcription factors. Moreover, qRT–PCR results demonstrated the high expression of *AcC3H1* and *AcREM14* in *Psa*-induced highly resistant hybrid lines. Ultimately, Overexpression of *AcC3H1* and *AcREM14* in kiwifruit enhanced disease resistance, and this was associated with upregulation of enzymatic activity and gene expression in the salicylic acid (SA) signaling pathway. Our study elucidates a molecular mechanism underlying disease resistance in kiwifruit and contributes to the advancement of research on kiwifruit breeding.

## Introduction

Kiwifruit is widely recognized as a nutritious food owing to its high vitamin C content and abundance of other essential nutrients [1]. China is the world’s major producer and distributor of kiwifruit and therefore possesses rich germplasm resources. *Actinidia chinensis* var. *chinensis* and *A. chinensis* var. *deliciosa* are among the most widely grown kiwifruit varieties [2, 3]. In particular, *A. chinensis* var. *chinensis*, including the well-known cultivar ‘Hort16A’ in New Zealand [4] and ‘Hongyang’ in China, has excellent quality and superior commercial value. Kiwifruit canker is a devastating disease that poses a severe threat to the cultivation and commercial production of kiwifruit. The main symptoms include leaf spots, rotten flowers, and mucus exudation, which can destroy the entire kiwifruit garden [5]. Kiwifruit canker disease is difficult to prevent and control because of its explosive and destructive nature and its propagation cycle within the plant. This disease has spread widely in areas where *A. chinensis* var. *chinensis* is cultivated, including China, Japan, Spain, New Zealand, Italy, and Turkey [6–10]. In China, kiwifruit canker mainly affects the central provinces, including Shaanxi and Sichuan [11, 12]. Currently, most *A. chinensis* var. *chinensis* are sensitive to *Pseudomonas syringae* pv. *actinidiae* (*Psa*) [13]. Thus, mining and screening this variety for resistance is critical for controlling canker disease.

In nature, plants regularly face threats from various pathogens, such as bacteria, fungi, and viruses. During co-evolution, plants and pathogens have always engaged in an arms race in which each organism strives to outcompete or evade the other. To accomplish this, plants have developed complex defense mechanisms to protect themselves from invaders. In response, pathogens have developed various strategies to avoid or destroy plant defense mechanisms and successfully infect the host [14]. Infection resistance in plants consists of both constitutive defenses, such as plant cuticular layers, cell walls, and preformed antimicrobial compounds, and induced defenses, which are triggered by the recognition of pathogen-associated molecular patterns and pathogen-secreted effectors [15]. To defend against pathogen attack, molecules such as calcium (Ca^2+^), reactive oxygen species (ROS), salicylic acid (SA), jasmonic acid (JA), ethylene, and flavonoids regulate disease resistance and defense signaling networks [16, 17].

Significant efforts have been made to identify kiwifruit cultivars that exhibit resistance to *Psa*. A single quantitative trait locus (QTL) for *Psa* resistance has been identified in linkage group 27 of *A. chinensis* var. *chinensis* ‘Hort16A’ using a high-density genetic map, and six minor QTL loci were identified in a resistant breeding parent based on a dense phenotyping technique [18]. Transcriptomic analysis of different kiwifruit varieties has indicated that both coding and non-coding regions in kiwifruit may be involved in responses to *Psa* [19]. Research has also shown that signal regulatory networks and metabolic pathways play essential roles in kiwifruit immunity. Changes in hormones, such as SA and JA, as well as secondary metabolites, including ROS, malondialdehyde (MDA), and superoxide dismutase (SOD), have also been found to be important indicators of plant resistance [20, 21]. It has been shown that JA accumulates in the sensitive line of *A. chinensis* var. *chinensis* ‘Hongyang’, but decreases in the resistant line *A. chinensis* var. *deliciosa* ‘Jinkui’ during *Psa* infection [22]. Song *et al*. showed that the SA-controlled signaling pathway genes *NPR1*, *TGA*, and *PR1* displayed higher expression in the resistant line *Actinidia eriantha* var. ‘Huate’ than in the sensitive ‘Hongyang’ [23]. These findings indicate that the SA and JA signaling pathways may be involved in the resistance response to *Psa*. In addition, kiwifruit regulates its metabolism to suppress infection, and *Psa* can enhance pathogenesis by manipulating the carbon/nitrogen metabolic pathway and sugar-mediated immunity [24].

The CCCH (C3H) type zinc finger transcription factor (TF) and B3 DNA-binding protein have been reported to be involved in the regulation of biotic and abiotic stresses in plants [25–29]. Silencing *GhC3H20* reduces the tolerance of cotton to salt stress. A yeast two-hybrid test identified two GhC3H20 interacting proteins (GhPP2CA and GhHAB1). GhC3H20 interacts with GhPP2CA and GhHAB1 to participate in the abscisic acid (ABA) signaling pathway and improve salt tolerance in cotton [30]. The *CARAV1* gene (B3 domain protein) has been reported to enhance the infection of transgenic *Arabidopsis thaliana* with *P. syringae* pv. *tomato* DC3000 and the activation of the *CARAV1* promoter is induced by *Bemisia tabaci*, SA, and ABA [31]. However, there are few reports on the two types of TF in kiwifruit. Additionally, there is limited research on the function of B3 DNA-binding proteins and C3H TFs, which requires further exploration.

To date, studies have demonstrated limited understanding of the mechanisms underlying kiwifruit resistance to canker diseases. In this study, we aimed to identify the traits associated with *Psa* resistance in *F*_1_ progeny derived from the same parental lines. We evaluated 44 *A. chinensis* var. *chinensis* hybrid lines and selected two *F*_1_ representative offspring that exhibited different sensitivities to *Psa*. We used RNA-seq to perform comparative transcriptomics of the two progenies, including differential gene expression analysis and weighted gene co-expression network analysis (WGCNA). This allowed us to identify multiple genes associated with resistance and susceptibility, as well as the associated pathways that correlate with *Psa* infection. Our study demonstrated that comparing kiwifruit germplasm resources with a consistent genetic background is highly productive in revealing disease resistance pathways. Our findings lay a foundation for the breeding of kiwifruits resistant to *Psa* infection.

## Results

### Selection of *F*_1_ kiwifruit hybrids with differential susceptibility to *Psa* infection

**Figure 1:**
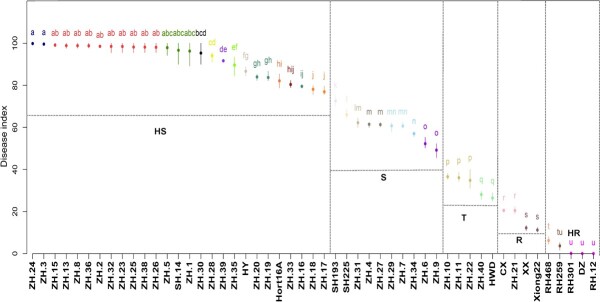
Kiwifruit leaf disease index evaluating *Psa* susceptibility of *F*_1_ offspring. Kiwifruit names with prefixes ZH, SH, and RH belong to 44 *A. chinensis* var. *chinensis* hybrid lines. *Actinidia chinensis* var. *deliciosa* ‘Xuxiang’ (XX, resistant), *A. chinensis* var. *deliciosa* ‘Cuixiang’ (CX, resistant), *A. chinensis* var. *chinensis* ‘Hongyang’ (HY, highly susceptible), *A. chinensis* var. *chinensis* ‘Hort16A’ (Hort16A, highly susceptible), *A. chinensis* var. *chinensis* ‘Xiong22’ (Xiong22, resistant), *A. chinensis* var. *deliciosa* ‘Hayward’ (HWD, tolerant) and *Actinidia macrosperma* var. ‘Dazi’ (DZ, highly resistant) were utilized as the seven controls. Different letters denote statistically significant differences (*P* < 0.05, Duncan’s multiple range test).

To identify traits associated with *Psa* resistance and to minimize contributions from different genetic backgrounds, we first generated kiwifruit *F*_1_ progeny by crossing *A. chinensis* var. *chinensis* ‘Xiong22’ (♂, *Psa*-resistant) with *A. chinensis* var. *chinensis* ‘Hort16A’ (♀, *Psa*-susceptible). We obtained 44 *F*_1_ offspring and tested their susceptibility to *Psa* infection using leaf disc assays. Based on the disease index (*D*) [32], we classified the 44 hybrid lines into four categories: high resistance (HR, *D* < 10), resistance (R, 10 ≤ *D* < 25), tolerance (T, 25 ≤ *D* < 40), susceptibility (S, 40 ≤ *D* < 65), and high susceptibility (HS, *D* ≥ 65). We noticed that most of the kiwifruit offspring lines were S or HS (35 out of 44), and there were four lines with HR phenotype, namely RH468, RH259, RH301, and RH12. Among them, RH12 was the line with the best phenotype (Fig. 1).

**Figure 2:**
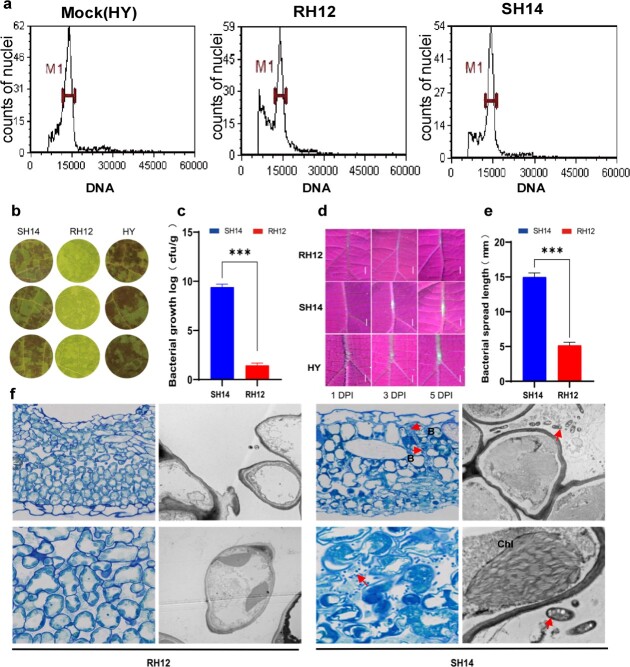
Comprehensive phenotypic analysis of *A. chinensis* var. *chinensis* hybrid RH12 and SH14 lines upon *Psa* infection. **a** Ploidy detection of RH12 and SH14 by flow cytometry. HY, *A. chinensis* var. *chinensis ‘*Hongyang’, a known diploid, was used as a control. **b** Leaf disc assay showing differences in *Psa* resistance between RH12 and SH14. HY, *A. chinensis* var. *chinensis* ‘Hongyang’, was used as a control, hereafter. **c** Quantitative measurement of *Psa*-infected RH12 and SH14 leaf discs shown in (**b**). ^***^P < 0.001 (Student’s two-tailed *t* test). **d** Bacterial growth in leaf veins at different time points under UV light. Scale bars represent 5 mm. **e** Infected vein length shown in (**d**). ^***^P < 0.001 (Student’s two-tailed *t* test). **f** Light and transmission electron micrographs of cross-sections of kiwifruit leaves (RH12 and SH14) infected with *Psa*. Chl, chloroplast.

To further confirm the observed resistance, we selected RH12 and a representative HS line, SH14, and performed several virulence tests. Both lines exhibited the same ploidy, as determined by flow cytometry (2*n* = 58 chromosomes) (Fig. 2a). Leaf disc assays consistently showed that SH14 was highly susceptible to the high-virulence *Psa* strain M228, whereas RH12 was significantly more resistant (Student’s *t* test*, P* < 0.001) (Fig. 2b and c). We also performed a leaf vein assay by inoculating *Psa* into kiwifruit veins through a puncture wound. To assess the dissemination of infection, we used an eGFP-labeled *Psa* M228 strain (Fig. 2b). As shown in Fig. 2d and e, at 5 days post-inoculation (dpi) the kiwifruit veins of the two lines showed significantly different pathogen expansion (Student’s two-tailed *t* test, *P* < 0.001). Light and transmitted light microscopy showed extensive colonization of pathogenic bacteria in the intercellular spaces of *Psa-*infected SH14 leaf tissues at 3 dpi. Further cytological observations using transmission electron microscopy revealed that infection with the pathogen led to cytoplasmic shrinkage and chloroplast disintegration in SH14. However, both the membrane structures remained relatively intact. In contrast, RH12 leaf tissues exhibited dense cytoplasm containing intact organelles and minimal bacterial distribution in the intercellular spaces (Fig. 2f). Overall, the results of these different virulence tests illustrate that we identified two *F*_1_ kiwifruit hybrids that were derived from the same parental lines but exhibited varying susceptibility to *Psa*.

### Kiwifruit lines with different disease susceptibilities exhibit distinct immune responses to *Psa* infection

After infection, plants typically regulate their metabolites to defend against pathogens [33]. It has been reported that, at the early stages of *Psa* infection, kiwifruit resistance depends on the activation of the antioxidant system [34]. To characterize the differences between the responses of RH12 and SH14 lines to *Psa* infection, we measured several aspects of the plant immune response during early-stage infection (0–24 h). RH12 demonstrated an earlier ROS burst, with a 1.59-fold higher H_2_O_2_ concentration than SH14 at 6 h post-inoculation (hpi) (Supplementary Data Fig. S2a). We found that MDA and hydroxyl ion (OH) levels in SH14 significantly increased at 6 hpi compared with those in RH12 (Supplementary Data Fig. S2b and d). The maximum OH content in SH14 (130.3 U μg^−1^ FW) was 1.72 times higher than that observed in RH12 at 24 hpi. However, no significant changes were observed in electrolyte leakage in RH12 and SH14 in response to pathogen infection (Supplementary Data Fig. S2c). In contrast, changes in proline (PRO) and glutathione (GSH) contents were more pronounced in RH12 (Supplementary Data Fig. S2e and f). PRO levels increased with infection time in both kiwifruit lines. However, higher PRO levels in RH12 than in SH14 were evident as early as 6 hpi. We also measured the activities of SOD and catalase (CAT), as these two molecules reflect the severity of *Psa* infection in kiwifruit leaves [35]. We found that CAT activity in the two lines significantly increased from 0 to 12 hpi, peaking at 12 hpi; however, CAT activity in SH14 was lower than that in RH12 at 12 hpi (Supplementary Data Fig. S2g). The RH12 and SH14 lines also varied in terms of their SOD activity. RH12 displayed an earlier increase in SOD activity, peaking (82.33 U g^−1^ FW) at 6 hpi, whereas SH14 reached its peak SOD activity at 12 hpi (Supplementary Data Fig. S2h). Additionally, we found that there was a higher accumulation of SA in SH14, ~2-fold higher than in RH12. However, JA was significantly induced in SH14, whereas it remained unchanged in RH12, suggesting that JA may mediate the susceptibility of kiwifruit to *Psa* infection (Supplementary Data Fig. S2i). Collectively, our analyses indicate that RH12 and SH14 kiwifruit lines exhibit different immune responses to *Psa* infection, which may contribute to their divergent resistance.

### Global transcriptome analysis of RH12 and SH14 upon *Psa* infection

To further investigate the mechanisms underlying the differential responses of RH12 and SH14 to *Psa* infection, we performed RNA sequencing (RNA-seq) on leaf discs of RH12 and SH14 infected with *Psa.* Infected samples were collected at four time points (0, 6, 12, and 24 hpi), based on a previous report [36]. On average, 84 020 044 high-quality reads were generated from each sample. Of these, 83.55% (RH12) and 88.61% (SH14) were mapped to the Hong Yang v3 genome (http://kiwifruitgenome.org/) (Supplementary Data Table S1). Additionally, principal component analysis revealed that RH12 and SH14 exhibited distinct patterns of gene expression migration over time during *Psa* infection (Supplementary Data Fig. S3).

**Figure 3:**
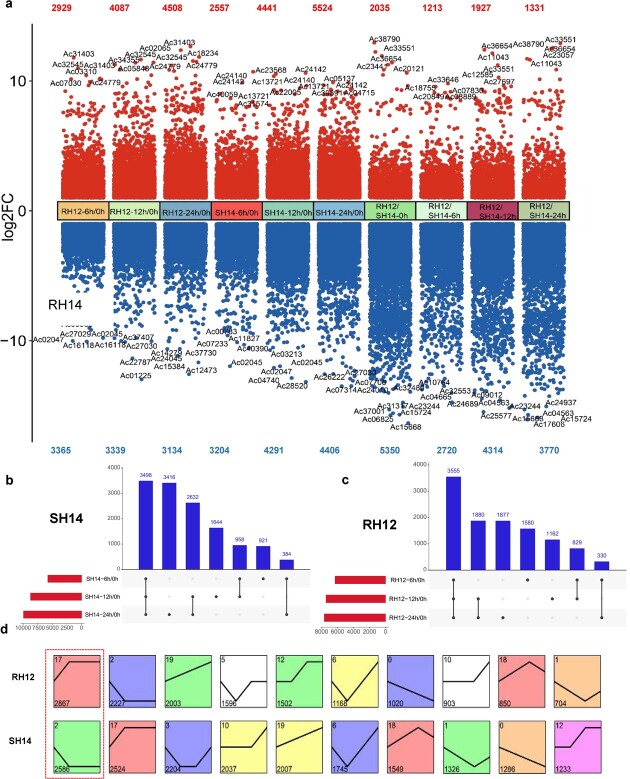
Differential gene expression analysis in *A. chinensis* var. *chinensis* hybrids RH12 and SH14 upon *Psa* infection. **a** Number of DEGs and highly expressed genes in RH12 and SH14. The criteria for screening DEGs were |log_2_fold change| > 1 and FDR < 0.01. FC, fold change. The gene identifiers of the top five DEGs in each comparison group are shown in the figure. RH12-6 h/0 h (RH12-12 h/0 h, RH12-24 h/0 h) represents the comparison between RH12 at 6 and 0 h (RH12 at 12 versus 0 h, RH12 at 24 versus 0 h). SH14-6 h/0 h (SH14-12 h/0 h, SH14-24 h/0 h) represents the comparison of SH14 at 6 versus 0 h (SH14 at 12 versus 0 h, SH14 at 24 versus 0 h). RH12/SH14-0 h (RH12/SH14-6 h, RH12/SH14-12 h, RH12/SH14-24 h) represents the comparison between RH12 and SH14 at 0 h (6, 12, and 24 h). **b**, **c** Significantly differentially expressed genes in RH12 and SH14. **d** STEM (Short Time-series Expression Miner) analysis of DEG expression trends in the two kiwifruit lines. In each profile, the line represents the expression trend of DEGs; the number above represents the number of profiles, and the bottom number represents the number of DEGs in each profile.

To identify differentially expressed genes (DEGs), we used two approaches simultaneously. First, we compared the genes expressed at 6, 12, and 24 hpi with those at 0 hpi for each kiwifruit line. We observed that, as the infection progressed, the number of upregulated genes increased in both lines. Interestingly, although the number of downregulated genes in RH12 decreased gradually during infection, the number increased over time in SH14 (Fig. 3a). SH14 had the largest DEG pool at 24 hpi compared with all 10 of the identified dynamic comparison groups, with 5524 upregulated genes and 4406 downregulated genes. Strikingly, *Actinidia39891.t1* and *Actinidia05137.t1* were among the highly upregulated genes, both of which were related to redox and flavonoid synthesis (Fig. 3a, Supplementary Data Table S2), indicating that these two genes may play a key role in disease resistance. We also compared the gene expression levels between the two lines at each time point. We identified 2035 DEGs with high expression levels in RH12 at 0 hpi and 1927 highly expressed DEGs at 12 hpi. Based on the 10 comparison groups (Fig. 3a), several of the top five most highly expressed genes were associated with disease resistance, such as the disease-related gene *Actinidia37001.t1*, a receptor kinase gene (*Actinidia11043.t1*), and the disease-related cell death gene *Actinidia33551.t1*.

#### Temporal trends and Kyoto Encyclopedia of Genes and Genomes analysis of differentially expressed genes

A comparison of gene expression in RH12 and SH14 at different time points revealed fewer DEGs in RH12 than in SH14 (11 013 versus 13 280). When we compared gene expression at 6, 12, and 24 hpi with expression at 0 hpi in each line, we observed that the numbers of DEGs at all three time points in RH12 and SH14 were approximately equal (3555 versus 3486). However, the largest number of DEGs was observed at 24 hpi (versus 0 hpi), when SH14 had 1539 more DEGs than RH12 (Fig. 3a–c).

To identify the pathways enriched for DEGs, we performed Kyoto Encyclopedia of Genes and Genomes (KEGG) analysis on the DEGs from 10 comparison groups (Fig. 3a) and identified the top 15 pathways (Supplementary Data Fig. S5). The plant hormone signal transduction pathway was enriched for DEGs in all 10 comparison groups. The pathways of flavonoid biosynthesis, phenylpropanoid biosynthesis, and plant–pathogen interactions showed a high enrichment of DEGs. Gene expression trends reflected the specific responses of kiwifruit to *Psa* infection. The results revealed opposite expression patterns of DEGs between the two lines during infection, such as profile17 and profile2, profile19 and profile0 (Fig. 3d). Among the profiles, module profile17 in RH12 showed an upward expression trend in 2867 genes (*P* < 0.05), whereas module profile2 in SH14 showed a downward trend in 2586 genes (*P* < 0.05). Gene Ontology (GO) analysis revealed that there were six common GO terms (response to external stimulus, plasma membrane, vacuole, cell wall, external encapsulating structure, and transferase activity) present in both profile2 (SH14) and profile17 (RH12) (Supplementary Data Fig. S4a and b). Moreover, expression analysis of the DEGs for these six GO terms revealed inconsistent expression patterns between RH12 and SH14 in profile17 (Supplementary Data Fig. S4c–h). In profile17, RH12 exhibited an upregulation trend among the DEGs, whereas SH14 showed diverse expression patterns with both upregulation and downregulation evident in the DEGs. Additionally, the DEGs in profile2 were downregulated in SH14, whereas only a few DEGs in RH12 were downregulated. However, significant differences in expression were primarily observed between RH12-0 h and SH14-0 h (Supplementary Data Fig. S4c–h).

#### Temporal and spatial expression of hormone signaling, flavonoid, and plant–pathogen pathway interactions differs between RH12 and SH14

According to KEGG enrichment analysis, we found that DEGs related to ‘plant hormone signal transduction’ and ‘flavonoid’ and ‘plant hormone signal transduction’ were significantly enriched (Supplementary Data Fig. S5). As hormone signal transduction plays an active role in plant immune regulation [37], we selected key genes involved in five major hormone-signaling pathways related to plant defense and examined their expression in RH12 and SH14 (Supplementary Data Fig. S6a). We identified 120 DEGs involved in the kiwifruit hormone signal transduction process (Supplementary Data Table S3a). As shown in Supplementary Data Fig. S6a, the expression patterns of DEGs in the ethylene signaling pathway differed between RH12 and SH14. A notable example is the JA pathway and we found that the JAZ-like gene (*Actinidia37726.t1*) was not significantly induced in either line, but its expression in SH14 was significantly lower than that in RH12 at 12 and 24 hpi. For the SA pathway, we found that the positive regulators of *TGA*-, *PR1*-, and *NPR1*-like genes (Supplementary Data Table S5) were induced by *Psa*, particularly *Actinidia39176.t2* (*NPR1*-like).

To further explore the DEGs involved in the interactions of SH14 and RH12 in the plant–pathogen interaction pathway, we obtained the genes in this pathway in kiwifruit by performing BLASTp comparison with *A. thaliana* (E-values <10^−3^). We identified 131 DEGs with adequate expression (FPKM <0.01) and several genes that appeared to respond to *Psa* infection (Supplementary Data Table S3b, Supplementary Data Fig. S6b). Notably, we found that two key components of the calcium signal transduction pathway, *CDPK* and *CaMCML*, showed similar expression trends in both kiwifruit lines, but their expression in RH12 was significantly higher than in SH14 (*P* < 0.05) (Supplementary Data Table S3b). In addition, we observed that the expression of the ROS regulator *RBOH* in RH12 was significantly higher than that in SH14 at 12 and 24 hpi; it is an important regulator of ROS burst and therefore resistance to pathogen infection, which was the original downregulation trend of *Actinidia28337.t1* (an *RBOH*-like gene) (Supplementary Data Fig. S6b).

Blastn analysis mapped 115 DEGs to the flavonoid pathway in RH12 and SH14 (Supplementary Data Table S3c, Supplementary Data Fig. S6c). Several DEGs in the phenylpropanoid biosynthesis pathway were annotated as key phenylalanine ammonia lyase (*PAL*)-like genes, which are essential for phenylpropanoid biosynthesis [38]. Of all the *PAL*-like genes, only six were significantly upregulated (*P* < 0.05) in RH12 (Supplementary Data Table S3c). We identified 16 *F3H*-like genes in the flavonoid pathway that play key roles in regulating the accumulation of flavonoid metabolites in plants [39]. In addition, we identified DEGs homologous to *TT5* (chalcone synthase) and chalcone isomerase-like (*CHIL*) genes, which synthesize pinocembrin, liquiritigenin, butin, and naringenin (Supplementary Data Table S3c, Supplementary Data Fig. S6c). These results suggest that the expression of flavonoid biosynthesis genes was highly upregulated in the resistant line RH12 in response to *Psa* stress.

### Co-expression network analysis provides insights into the genes contributing to *Psa* resistance

WGCNA was used to identify highly correlated gene clusters and link them to biological traits [40]. Rather than concentrating on a single gene or isolated biomarker, WGCNA modularly investigates co-expressed genes and extracts intramodular hub genes from system networks, which increases the sensitivity of recognizing important targets for biological regulation [41]. To further identify the key genes involved in pathogen resistance in RH12 and SH14, we performed WGCNA. Based on a power value of 16 (Supplementary Data Fig. S7), we grouped 23 samples and 30 344 DEGs into 20 modules (Supplementary Data Fig. S8a). The samples at different time points had different degrees of association with the traits (Supplementary Data Fig. S8b). Likewise, association analysis revealed relationships between the traits of interest and the specific gene modules (Supplementary Data Fig. S8c). The number of genes varied significantly among the modules. The most extensive module (MEturquoise) possessed 5800 genes, whereas the smallest module (MEroyalblue) contained only 32 genes. Genes in each module were considered co-expressed and functionally related. Because highly correlated modules (red squares) are likely to be involved in similar biological processes, four highly trait-specific modules were further analyzed (Supplementary Data Fig. S8c). We found that the MEblack module (R > 0.8) was highly correlated with GSH and R.S (R, resistant; S, susceptible), whereas the MEgreen module (R > 0.64) was highly positively correlated with the H_2_O_2_, PRO, electrolyte, GSH, and OH pathways. In addition, MEturquoise (R > 0.77) positively correlated with MDA, electrolyte, and OH. The MEpink module (R < −0.73) was highly negatively correlated with R.S (Supplementary Data Fig. S9). Overall, the correlation between genes in the modules and their corresponding traits suggests that genes within a module may have similar functions that can be related to phenotypes of interest.

#### Regulatory networks of the resistance-related modules

**Figure 4:**
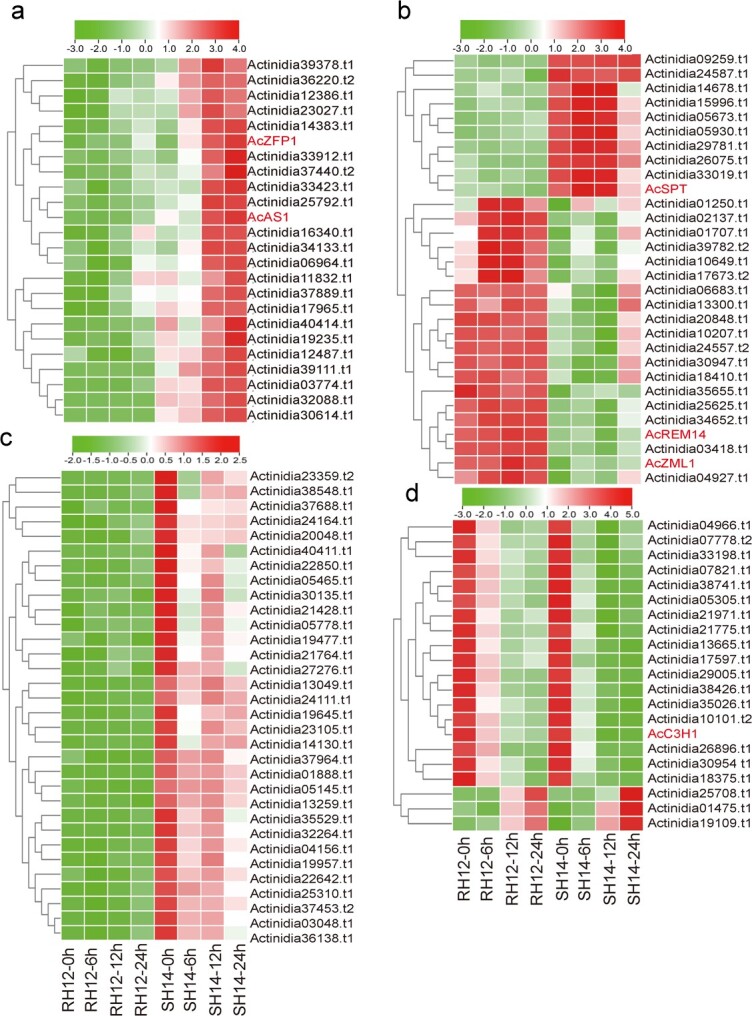
Heat maps showing the top 5% hub genes in the four WGCNA modules. **a** Expression patterns of 24 hub genes in the MEturquoise module. **b** Expression patterns of 30 hub genes in the MEblack module. **c** Expression patterns of 32 hub genes in the MEpink module. **d** Expression patterns of 21 hub genes in the MEgreen module.

To further investigate hub genes in the WGCNA modules, we screened 592 (weight > 0.15), 636 (weight > 0.15), 423 (weight > 0.15), and 483 (weight > 0.35) genes from the four modules. We analyzed the gene expression profiles of the four modules and found that the gene expression in each module was unique. In the MEgreen module, the expression trends for RH12 and SH14 were the same, whereas in the MEblack module RH12 and SH14 displayed opposing expression patterns (Supplementary Data Fig. S10a and b). In comparison, the MEpink and MEturquoise modules had similar expression patterns; the genes in the modules were highly expressed in SH14 (Supplementary Data Fig. S10a and b). To detect critical hub genes, we performed cytoHubba analysis to efficiently screen for genes. We defined hub genes by selecting the top 5% of genes with the highest degree (most edges) in each module (Fig. 4) and identified a subset of 107 hub genes. Of the hub genes in the MEturquoise module (Fig. 4a), *Actinidia37889.t1* (β-glucosidase) had the highest degree (69 edges) and may be involved in regulating processes such as lignification of plant cell walls and defense responses of plants to adversity [42, 43]. We also found that several hub genes were DEGs involved in the activation of cell wall lignification hormones and release of aromatic compounds. In the MEblack module, we found critical hub genes involved in salt resistance and plant cell wall synthesis (*Actinidia33019.t1* and *Actinidia01250.t1*), and encoding M3KP1 (*Actinidia14678.t1*) and protein kinases (*Actinidia04156.t1* and *Actinidia27276.t1*) that regulate ROS. Intriguingly, critical hub genes in the MEblack (Fig. 4b) and MEpink (Fig. 4c) modules were expressed in opposite directions. We identified 21 critical hub genes in the MEgreen module (Fig. 4d), and the highest-degree hub gene was annotated as *Actinidia38741.t1* (protein-enhanced disease resistance 2) (40 edges).

#### Functional annotation of hub genes

To better understand the function of traits highly correlated with the modules, the hub genes of the four modules were subjected to GO analysis (*P* < 0.05). We found that the genes co-expressed in the MEgreen module (electrolyte- and H_2_O_2_-specific modules) (Supplementary Data Fig. S10c, Supplementary Data Table S6a) were related to ‘RNA binding’, ‘generation of precursors and energy’, and ‘nucleoplasm’. Notably, the biological processes involved in ‘metabolic process’ were highly enriched. In the MEblack module (Supplementary Data Fig. S10d**,**Supplementary Data Table S6b), genes were enriched for ‘lipid metabolism processes’, ‘lysosomes’, and ‘cell cycle’ functions. The genes in the MEpink module (Supplementary Data Fig. S11c, Supplementary Data Table S6c) were mainly involved in ‘cell wall’, ‘cytoskeleton’, and ‘response to biotic stimulus’ roles. In comparison, genes in the MEturquoise module were enriched in ‘chloroplast’, ‘thylakoid’, and ‘photosynthesis biological processes’ (Supplementary Data Fig. S11d, Supplementary Data Table S6d). In summary, we identified hub genes associated with disease resistance.

### Identification of the potential transcription factors that regulate kiwifruit resistance to *Psa* infection

TFs are activated after a series of signal transduction events when plants sense invading pathogens. Activated TFs specifically interact with *cis*-acting elements in the corresponding gene promoters in the nucleus, thereby activating the transcriptional expression of defense response-related genes [44]. We specifically investigated the TFs in the modules and identified 661 TFs, accounting for 29.35% of all kiwifruit TFs (Supplementary Data Table S5). Most TFs were enriched in the MEgreen module (325), whereas the MEpink module was least enriched (52). We found that the largest number of TFs (71) belonged to the NAC family, accounting for 57.25% (71/124) of the NAC TFs in kiwifruit. Other identified TFs belonged to the bHLH, MYB, C2H2, and ERF families, accounting for 20.00, 31.67, 25.69, and 21.88% of the total TFs in their respective families (Supplementary Data Table S5). We further screened the co-expression network using Cytoscape and identified 115 TFs that were displayed in the co-expression network of the four modules.

To identify the TFs critical for kiwifruit defense against *Psa* infection, we constructed a network using cytoHubba analysis. We discovered six (*AcSPT*, *AcZML1*, *AcREM14*, *AcC3H1*, *AcAS1*, and *AcZFP1*) high-degree (top 5%) TFs in four modules (Supplementary Data Table S6). Further analysis revealed that these six TFs may interact with several critical resistance genes (Supplementary Data Table S8). Notably, *AcSPT* and *AcMAPKKK1* (mitogen-activated protein kinase kinase kinase1) (*Actinidia14678.t1*) were closely connected. *AcZFP1* and *AcLRR-RLP* (*Actinidia19235.t1*) may have an interactive regulatory relationship (Supplementary Data Fig. S10e and f) (Supplementary Data Fig. S11e and f). In addition, *cis*-acting element analysis of the six TFs revealed a variety of hormone regulatory sites. For example, many response elements related to adversity and involved in ‘MeJA-responsiveness’ and ‘abscisic acid’ were identified in *AcSPT*. *AcREM14* also contained a gibberellin-responsive element (Supplementary Data Table S7). In summary, through co-expression analysis, we found six TFs likely to play essential roles in defending against bacterial canker disease of kiwifruit.

To verify the gene expression profiles obtained from RNA-seq, we selected 20 genes, including those involved in important signaling pathways as well as predicted hub genes, and performed quantitative PCR analysis, which indicated that the relative gene expression of these selected genes was generally consistent with the transcriptomic data (Supplementary Data Fig. S12).

**Figure 5:**
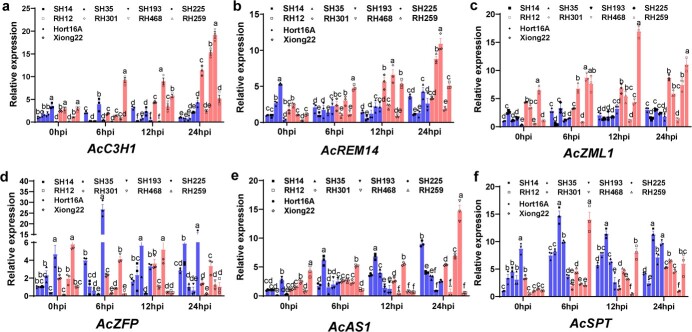
qRT–PCR was performed to determine the expression levels of the six TFs in the eight samples. Among them, ‘Hort16A’, SH14, SH35 (ZH35), SH193, and SH225 were highly susceptible (HS) materials, RH12, RH301, RH468, and RH259 were highly resistance (HR), and ‘Xiong22’ was resistant (R). Data are presented as mean ± standard deviation and each assay was repeated three times. Different letters indicate statistical significance (Duncan’s multiple range test, *P* < 0.05).

To investigate whether the expression of the six core TFs is associated with resistance of diploid kiwifruit to bacterial canker disease, we infected four HS kiwifruit materials (SH14, SH35, SH193, and SH225) and four HR materials (RH12, RH301, RH468, and RH259) (Fig. 1, Supplementary Data Fig. S1), which belong to the same hybrid diploid population as *Psa*-M228, and their expression was analyzed by qRT–PCR. Among them, ‘Hort16a’ and ‘Xiong22’ were used as the positive and negative controls, respectively. The experimental results showed that three TF genes (*AcC3H1*, *AcZML1*, and *AcREM14*)were highly expressed in the HR varieties. *AcZML1* and *AcC3H1* both showed a high expression trend induced by *Psa* in three HR materials (RH12, RH259, and RH468), whereas *AcREM14* showed a significantly high expression trend in three HR materials (RH12, RH259, and RH301) (*P* < 0.05). Based on these results, we speculated that the increase in the expression levels of three TF genes (*AcC3H1*, *AcZML1*, and *AcREM14*) may be a key factor in kiwifruit disease resistance (Fig. 5).

### *AcC3H1* and *AcREM14* are important regulators of disease resistance

**Figure 6:**
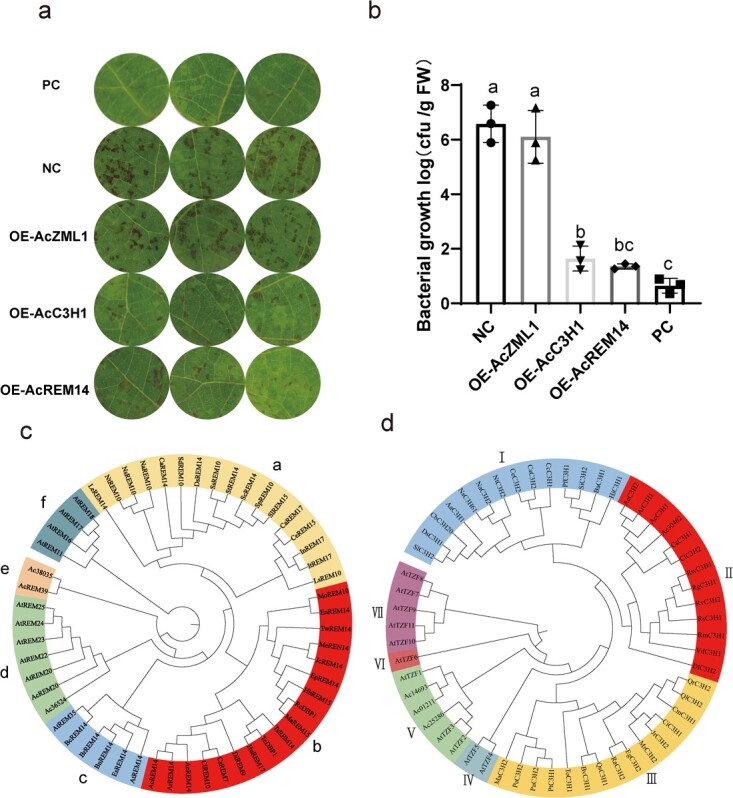
Fig. 6. Functional identification of three core transcription factors, AcC3H1, AcZML1, and AcREM14, and evolutionary analysis of AcREM14 and AcC3H1. **a** Phenotypic observation of overexpressing leaf discs after 5 days of *Psa* infection. PC, the positive control, refers to the transient overexpression of the empty vector pCAMBIA1302 in ‘Xiong22’, followed by inoculation with *Psa*. Similarly, transient overexpression of the empty vector pCAMBIA1302 in kiwifruit ‘Hort16A’ leaves, followed by inoculation with *Psa*, served as a negative control (NC). **b** Biological content of bacteria M228 (*Psa*) in leaf discs. Three independent biological replicates were used for each experiment. The least significant difference method was used for statistical analysis. Different letters indicate statistical significance (*P* < 0.05). **c**, **d** Systematic evolutionary analysis of homologous proteins of (**c**) AcREM14 and (**d**) AcC3H1 in different species. The letters a-f and the numbers I-VI respectively represent the grouping of homologous proteins of AcREM14 and AcC3H1. MEGAX (https://www.megasoftware.net/dload_mac_beta) was used to construct phylogenetic trees. The neighbor-joining method was used with a bootstrap value of 1000. The evolutionary gene names were based on the National Center for Biotechnology Information (NCBI) (https://www.ncbi.nlm.nih.gov/) annotations, and the corresponding gene IDs for the simplified gene names are shown in Supplementary Data Table S11.

To validate the disease resistance functions of the three TFs in kiwifruit, we individually constructed overexpression vectors for each TF and performed transient transformation experiments in kiwifruit. Overexpression of *AcREM14* and *AcC3H1* significantly reduced the symptoms in kiwifruit leaf discs (Fig. 6a). We then used qRT–PCR to confirm that all three genes were upregulated to varying degrees in ‘Hort16A’ (Supplementary Data Fig. S13a). Next, we used the plate-coating method to count the bacteria on four overexpressing leaf discs and found that bacterial colonization was significantly reduced in the discs treated with OE-*AcREM14* and OE-*AcC3H1*, which matched the statistical analysis of kiwifruit incidence (Fig. 6b, Supplementary Data Fig. S13b), indicating that *AcREM14* and *AcC3H1* can enhance the ability of kiwifruit leaves to resist *Psa*.

To study the evolutionary characteristics of AcREM14 and AcC3H1, homologous phylogenetic trees were constructed for these two proteins in different species. The study found that, among the homologous proteins in group b (Fig. 6c), AcREM14 had the highest number (18), accounting for 37.5% of the total homologous proteins (Fig. 6c). Multiple sequence alignments showed that AcREM14 had three typical B3 DNA-binding domains (Supplementary Data Fig. S14). The variability of REM14 homologous proteins was greater in kiwifruit and other species; however, the AcREM14 sequence was relatively conserved in kiwifruit, suggesting that this protein may be unique and plays a special role in kiwifruit. In contrast to AcREM14, AcC3H1 contained homologous proteins in multiple species (Fig. 6d, Supplementary Data Fig. S15) and its sequence variation was small in both kiwifruit and other species, indicating that this protein is functionally conserved. We also cloned the coding sequences of AcREM14 and AcC3H1 from RH12 and SH14 and translated them into proteins for comparison. The results showed that there were individual amino acid mutations in the sequences of these two proteins in RH12 and SH14, but no large-fragment deletions were observed (Supplementary Data Figs S14 and S15). Furthermore, through a comparative analysis of the promoter regions, we found that both AcREM14 (Supplementary Data Fig. S16a) and AcC3H1(Supplementary Data Fig. S16b) had base-point mutations in RH12 and SH14.

### Overexpression of *AcREM14* and *AcC3H1* affects the physiological characteristics of kiwifruit

**Figure 7:**
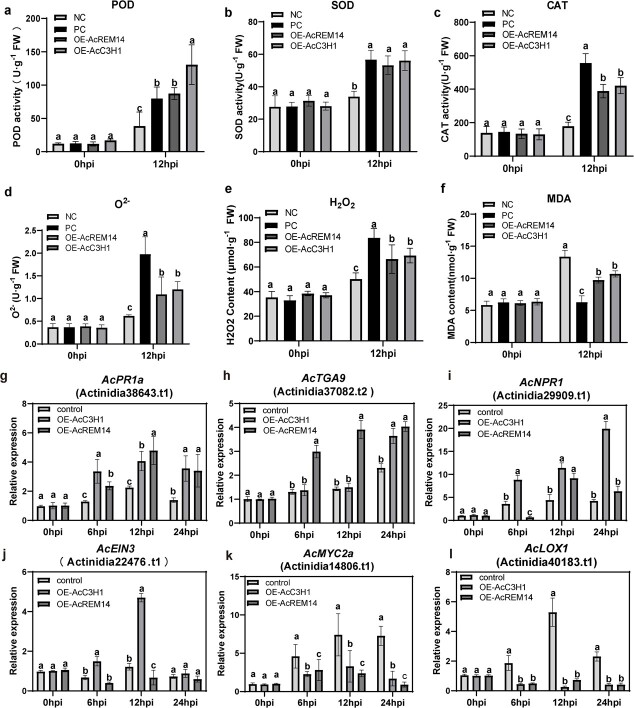
Physiological changes in kiwifruit leaves with transient overexpression (*AcREM14* and *AcC3H1*) under *Psa* infection. **a**–**c** Measurement of POD, SOD, and CAT activity. **d**, **e** Levels of superoxide anion (O^2−^) and H_2_O_2_. **f** MDA content. **g**–**i** Expression of defense genes *AcPR1a* (pathogenesis-related protein 1), *AcTGA9* (TGACG motif-binding factor), and *AcNPR1* (non-expressor of pathogenesis-related gene 1) in the SA signaling pathway, as detected by qRT–PCR. **j** Relative expression of defense marker genes in the ethylene pathway, *AcEIN3* (ethylene-insensitive 3). **k**, **l** Relative expression of defense genes *AcLOX* (lipoxygenase 1) and *AcMYC2a* (myelocytomatosis proteins 2) in the JA pathway. PC, the positive control, transiently overexpressed the empty vector pCAMBIA1302 in ‘Xiong22’, followed by inoculation with *Psa*. Similarly, transient overexpression of the empty vector pCAMBIA1302 in kiwifruit ‘Hort16A’ leaves, followed by inoculation with *Psa*, served as a negative control (NC). OE-AcREM14 refers to transient overexpression of *AcREM14* in ‘Hort16A’ leaves, whereas OE-AcC3H1 refers to transient overexpression of *AcC3H1* in ‘Hort16A’ leaves. Each experiment was conducted in triplicate. Different letters indicate statistical significance, *P* < 0.05 (Duncan’s multiple range test).

The effects of *AcREM14* and *AcC3H1* overexpression on the physiological characteristics of kiwifruit in response to bacterial canker disease were further explored. To achieve this, we transiently overexpressed *AcC3H1* and *AcREM14* genes in ‘Hort16A’ leaves. After 48 h of overexpression, plants were inoculated with *Psa*. The results indicated increased activities of defense enzymes (POD, SOD, and CAT) in kiwifruit leaf discs (Fig. 7a–c). Moreover, the transient overexpression of these two genes significantly promoted the burst of ROS in kiwifruit leaves (*P* < 0.05) (Fig. 7d and e). Notably, AcREM14 overexpression had a more significant effect than *AcC3H1* on promoting this burst (*P* < 0.05) (Fig. 7d and e). Additionally, MDA content significantly decreased compared with that in the control (Fig. 7f), suggesting that *AcC3H1* and *AcREM14* can alleviate membrane damage in kiwifruit leaves and enhance disease resistance. Furthermore, we investigated the expression of defense genes in the SA, JA, and ethylene pathways in leaf discs overexpressing *Psa*. qRT–PCR analysis revealed varying degrees of defense gene activation (*AcPR1a*, *AcTGA09*, and *AcNPR1*) in the SA pathway (Fig. 7g–j). In contrast, overexpression of *AcC3H1* alone may activate EIN3-mediated disease resistance in the ethylene pathway (Fig. 7k), indicating a potential role for AcREM14, primarily in the SA pathway. Remarkably, overexpression of *AcREM14* and *AcC3H1* resulted in a significant decrease in *AcLOX1* gene expression in the JA pathway at 6, 12, and 24 hpi (Fig. 7l). This suppression suggested that the downregulation of *AcLOX1* and *AcMYC2a* gene expression may hinder JA synthesis and subsequently weaken the sensitivity of kiwifruit to *Psa.*

## Discussion

In this study, we evaluated the resistance of 44 *F*_1_*A. chinensis* var. *chinensis* hybrids. Most *A. chinensis* var. *chinensis* lines were highly susceptible to *Psa* infection, which may be because of their low ploidy (2*n*). We identified the diploid *A. chinensis* var. *chinensis* line RH12, which showed high resistance to kiwifruit bacterial canker disease. Our comparative transcriptome analysis of two *F*_1_ hybrids with the same ploidy (2*n*) derived from the same parental lines but with different *Psa* resistance (RH12, high resistance; SH14, high susceptibility) largely reduced the contribution of genetic background to phenotypic differences. The utilization of these two lines facilitated our exploration of the genetic and regulatory mechanisms underlying the interactions between kiwifruit and *Psa*. Thus far, all previous comparative studies have sought to identify kiwifruit defense genes compared with kiwifruit with different genetic backgrounds. For instance *A. chinensis* var. *chinensis* ‘Hongyang’ versus *Actinidia eriantha* var. ‘Huate’ [23], and *A. chinensis* var. *deliciosa* and *Actinidia arguta* [45]. Variations across these genetic backgrounds present significant challenges in determining resistance mechanisms. In this study, we investigated *Psa* resistance-related traits in two *F*_1_ hybrids derived from the same parental lines, which was highly advantageous.

In our study, hormone signaling, flavonoid, and plant–pathogen interaction pathways were highly enriched by KEGG, indicating that these three pathways specifically play a role in the interaction between *Psa* and kiwifruit. A growing body of evidence suggests that the flavonoid pathway plays a vital role in plant adaptation to abiotic and biotic stress [46]. In the *Psa*-susceptible SH14 line, PAL-like genes were highly expressed at all four time points. Recent studies have shown that PALs are involved in the interactions between nucleotide-binding and leucine-rich repeat (NLR) proteins, as well as RNA-binding proteins that mediate broad-spectrum resistance in rice [47].

It was previously reported that inhibition of the ethylene and JA pathways occurs early after *Psa* infection [34], but this was not observed in our study. We believe that the innate (rather than *Psa*-induced) high expression in kiwifruit contributes more to resistance. In the ethylene pathway, SnRK2.8, a conserved kinase in *Arabidopsis*, phosphorylates the *P. syringae* effector protein AvrPtoB, thereby increasing virulence and reducing defenses in *Arabidopsis* [48]. In contrast, the expression patterns of SnRK-like genes in RH12 and SH14 showed opposite trends. Previous studies have shown that the expression of genes homologous to known resistance genes tends to be positively regulated in response to infection in resistant varieties, whereas these same genes are expressed at lower levels in response to infection in susceptible varieties [22]. In general, DEGs of the SA pathway have been widely reported to be involved in resistance to *P. syringae* [49]. Our investigation revealed that at 12 hpi with *Psa*, both JA and SA significantly accumulated in the susceptible varieties SH14 and ‘Hort16A’, whereas no significant change was observed in RH12 and ‘Xiong22’. (Supplementary Data Fig. S2). These findings suggest that JA plays a role in suppressing kiwifruit resistance to *Psa*. Subsequently, qRT–PCR analysis was conducted to assess the expression of marker genes involved in the JA (*AcAOC2*, *AcMYC2a*, *AcCOI1*, *AcAOS1*, *AcLOX1*, and *AcPR-4*) and SA (*AcNPR1*, *AcTGA04*, *AcTGA09*, and *AcPR1a*) pathways (Supplementary Data Fig. S12). Remarkably, the results were consistent with the trends observed in the transcriptome analysis. Furthermore, we identified a positive correlation between SA and JA synthesis and specific genes in kiwifruit. For instance, *AcPR1a* was upregulated in both RH12 and SH14 but showed a higher expression trend in SH14, which is consistent with the higher content of SA in SH14. Upregulation of the marker gene *AcMYC2a* in the JA synthesis pathway significantly promoted JA synthesis in SH14.

WGCNA has been frequently used to study plant interactions with pathogenic bacteria [50–52]. Wu *et al*. [53] constructed a co-expression network by WGCNA, which enabled the identification of a total of 5010 DEGs in 15 co-expression gene modules, and 4 modules showed significant association with smut resistance. The researchers identified 38 hub genes by calculating their connectivity within the corresponding network [50]. Here, we used WGCNA to correlate the expression profiles of RH12 and SH14 with the virulence phenotype and identified four highly correlated modules. According to REVIGO redundancy removal analysis, lipid metabolic processes are associated with disease resistance in kiwifruit. Our results support the conclusion that kiwifruit bacterial canker is associated with membrane lipid damage during *Psa* infection and that the lipid transfer protein StLTP10, a disease-resistance protein, enhances potato resistance to late blight fungus [44]. Using WGCNA, we narrowed down the number of critical hub genes involved in plant–pathogen interactions. By constructing a co-expression network, we identified several genes related to redox in hub genes with high connectivity (top 5%). Pathogen infection can cause plant lipid peroxidation and accumulation of ROS owing to the attack on membrane unsaturated fatty acids, which results in MDA accumulation. Accumulation of H_2_O_2_ can also trigger an increase in the concentration of intracellular calcium. Furthermore, the study observed no significant differences in the electrolyte leakage test between the resistant lines (‘Xiong22’ and RH12) and susceptible lines (‘Hort16A’ and SH14). This could be attributed to the relatively intact cell membranes in RH12 and SH14 (Fig. 2f), resulting in less leakage of intracellular molecules. Additionally, it may be associated with other potential disease resistance factors. For example, the activation of the immune genes *AcPR1a* and *AcPR-4* (Supplementary Data Fig. S12) may have a more significant impact on determining resistance. These genes are responsible for initiating plant defense responses against pathogens. The synthesis of defense compounds, including MDA, SOD, H_2_O_2_, and GSH (Supplementary Data Fig. S2), may also have a significant influence on disease resistance. These compounds are involved in various defense mechanisms, such as the inhibition of pathogen growth and mitigation of oxidative stress induced by *Psa*.

TFs have been reported to play crucial roles in regulating defense-related signaling pathways in response to pathogen infection [54]. It has been shown that the MYB, NAC, bHLH, and C2H2 TFs are indispensable for plant defense against *P. syringae* [55]. Our study showed that the six hub TFs (top 5%) have several *cis*-elements that respond to signals such as low temperature, hormones, and drought stress, indicating the importance of TFs involved in canker resistance. We identified *AcAS1*, a typical R2R3-MYB TF in the MEturquoise module, which was induced by *Psa* in the susceptible line SH14 (Supplementary Data Table S8). Similar to *AcAS1*, the other two TFs, *AcC3H1* and *AcZFP1*, belong to the zinc finger (CCCH-type) family of proteins. This study found that C3H-type zinc finger TFs can enhance plant resistance to pathogens by regulating the antioxidant response of plants. In *Arabidopsis*, the homologous genes of *AcC3H1*, *AtTZF1* (Tandem CCCH Zinc Finger 1), *AtTZF2* (Tandem CCCH Zinc Finger 2), and *AtTZF3* (Tandem CCCH Zinc Finger 3) (Fig. 6d) participate in the ABA/JA signaling pathway and regulate abiotic stress responses such as cold and salt stress [56–58]. However, there are currently no reports on the role of AcC3H1 in disease resistance. Our study demonstrated, for the first time, that AcC3H1 plays a role in resistance to bacterial canker disease in kiwifruit. We also found that *AcC3H1* is highly expressed in HR materials of *A. chinensis* and is highly associated with proteins such as ‘calcium-binding protein’ (Actinidia29005.t1) and ‘plant intracellular Ras-group-related LRR protein 7’ (Actinidia35026.t1) in the CytoHub network, which may be key to its involvement in disease resistance. Our hypothesis was also confirmed by transient overexpression experiments, which showed that *AcC3H1* and *AcREM14* (plant-specific B3 DNA-binding domain proteins) could enhance kiwifruit resistance to *Psa*.

Comparing the sequence alignment of *AcC3H1* and *AcREM14* in RH12 and SH14, differences were found in both the protein sequence and the promoter region, with significant variations mainly observed in the promoter region. Variations (point mutations and deletions) in the base positions of the promoter region in SH14 and RH12 may be key factors leading to expression discrepancies in these two TFs in response to *Psa* infection, particularly in resistant and susceptible cultivars, where the genes are expressed at higher levels in resistant cultivars. Additionally, among these two factors, only C3H1 has highly conserved homologous proteins in other species, whereas REM14 appears to have evolved in kiwifruit, displaying significant differences from other known homologous proteins. Further exploration is required to determine whether kiwifruit-specific AcREM14 has other functions. Physiological and qRT–PCR analyses revealed that the overexpression of these two genes induced a significant surge in ROS in kiwifruit and concurrently upregulated gene expression in the SA pathway. Therefore, we deduced that these two TFs play pivotal roles in modulating the SA pathway. However, a more detailed understanding of the precise mechanisms underlying disease resistance pathways requires further investigation using molecular techniques. With emerging genome-editing technologies, it will be of great interest to verify the function of these TFs in the future.

### Conclusions

In this study we selected two kiwifruit *F*_1_ progenies that displayed differing susceptibilities to *Psa* infection and performed comparative transcriptome studies of the two lines to identify the traits responsible for *Psa* resistance. Our results showed that 6 hpi was the critical time point for *Psa* infection, and both resistant and sensitive kiwifruit lines mounted a comprehensive immune response to *Psa* infection. To decipher the correlation between the groups of DEGs and several phenotypes related to pathogen infection and resistance, we applied WGCN and REVIGO analyses to unravel the core genes related to kiwifruit canker susceptibility. Strikingly, we found that three core TF genes (*AcC3H1*, *AcZML1*, and *AcREM14*) were induced by *Psa* and showed a high expression trend in the four disease-resistant varieties. Finally, we validated that *AcREM14* and *AcC3H1* could enhance kiwifruit resistance to *Psa* through overexpression experiments. Our study provides insights into the molecular mechanisms underlying *A.* var. *chinensis* resistance to *Psa* infection and support kiwifruit breeding research.

## Materials and methods

### Plant materials and leaf disc assay

Kiwifruits were routinely grown in a greenhouse (34°15′49″ N, 108°3′47″ E) at Northwest A&F University. For leaf disc assays, *Psa* M228 was cultured in Luria–Bertani (LB) medium at 25°C for 12–16 h until the OD_600_ reached 0.6. To obtain kiwifruit leaf discs, fresh kiwifruit leaves of similar size and shape were obtained, rinsed with sterile water, and soaked in 70% ethanol for 30 s. Next, the leaves were submerged in 2% sodium hypochlorite solution for 3 min and 70% ethanol for 20 s. Finally, the leaves were rinsed four times with sterile water and dried by tissue blotting. For each test, 100 leaf discs were excised using a 10-mm diameter cork borer. To establish *Psa* infection, leaf discs were placed in a test tube containing 1 × 10^5^ cells/ml of *Psa* [suspended in phosphate-buffered saline (PBS) buffer, pH 6.8]. The tubes were then vacuumed for 5 min in a desiccator three times until the floating leaves sank to the bottom of the tube. After vacuuming,16 leaf discs were placed on a water agar plate and incubated at 16°C with a 16-h light/8-h dark photoperiod for 0, 6, 12, or 24 h, depending on the time point. Freshly completed *Psa*-inoculated kiwifruit leaf discs were sampled, quickly frozen in liquid nitrogen, and marked at 0 hpi. Three biological replicates (three leaf disc plates) were assayed for each kiwifruit line at each time point. Infection symptoms on leaf discs were normally observed 5 dpi. For molecular analysis, the samples were quickly frozen in liquid nitrogen after harvesting and stored at −80°C for future use. To quantify the number of *Psa*-infected leaf discs, 0.2-g leaf discs at different time points were transferred to tubes containing 2.0 ml 0.9% NaCl and gently ground with quartz sand. Subsequently, the suspensions were subjected to a series of dilutions (10^0^, 10^−1^, 10^−2^, and 10^−3^), and 100 μl of each sample was plated onto the LB medium and cultured at 28°C for 72 h. After colonies appeared, their numbers were counted. Assays for each infected sample were repeated at least three times.

### Leaf vein assay

Leaf vein assays were performed according to a previously described method [59], with slight modifications. Briefly, kiwifruit leaves of similar sizes were rinsed with sterile water, soaked in 0.6% sodium hypochlorite solution for 5 min, and rinsed three times with sterile water. After drying by tissue blotting, petiole bases were wrapped in absorbent cotton. Wounds were punctured at the main vein, 1–2 cm from the petiole, and 10 μl of eGFP-labeled M228 (grown at OD_600_ = 0.1, and diluted to 1 × 10^8^ cells/ml) bacterial suspension was dropped onto the wound. After inoculation, the leaves were incubated under the following conditions: photoperiod light/dark 16/8 h; day and night temperature 16/4°C; and relative humidity 95%. The areas of pathogen expansion were visualized and measured by fluorescence stereomicroscopy (MZ10F, Leica, Germany).

### Transmission electron microscopy

The samples for transmission electron microscopy (TEM) were prepared as previously described [60]. Briefly, kiwifruit leaf discs at 5 dpi were excised into 1 mm × 1 mm × 3 mm pieces and fixed in 5.0 ml 4% (v/v) glutaraldehyde solution at 4°C overnight. Next, the leaf pieces were rinsed with PBS (pH 6.8) for 2 h, dehydrated with different concentrations of ethanol, and dried in a carbon dioxide critical-point dryer (Emitech, UK). Ultrathin sections (85 nm thick) were prepared for TEM using a UC7 ultra-thin sectioning machine (Leica, Germany) and placed on a copper mesh. After double staining with uranyl acetate and lead citrate, the samples were imaged using an HT-7700 transmission electron microscope (Hitachi, Japan) at 80.0 kV.

### RNA isolation and sequencing

Total RNA was extracted using a Trelief RNAprep Pure Plant Kit (TSP412, Tsingke), according to the manufacturer’s instructions. RNA quality was examined using 1.0% agarose gel and NanoDrop. RNA-seq libraries were prepared by Biomarker Biotechnology (Beijing, China). cDNA synthesis was performed using Phusion DNA polymerase, and 450-bp target fragments were selected for the library. An Illumina RNA library preparation kit was used for end repair, poly(A) addition, and adaptor ligation. Libraries were sequenced on an Illumina NovaSeq 6000 sequencing platform to obtain 150 bp paired-end reads. Read quality was checked using a Qsep400 Bio-Fragment Analyzer. See Supplementary Data Table S1 for the RNA-seq metrics.

### Differentially expressed gene and Gene Ontology enrichment analysis

To align RNA-seq reads to the genomes, the quality of the raw reads was first assessed using Trimmomatic v.0.39 [61] and fastp [62]. The filtered reads were aligned to the kiwifruit reference genome (http://kiwifruitgenome.org/) using HISAT2 v2.1.0 [63]. Transcript assembly was conducted using StringTie v1.3.3b [50] and read counts were calculated using the built-in prepDE.py script. The DEseq2 package v1.16.1 [64], was used to screen for transcripts with absolute expression fold-changes >2 and *P* < 0.05. For GO enrichment analysis, coding sequences were annotated using Blast2GO [65] based on the Swiss-Prot database (https://www.expasy.org/resources/uniprotkb-swiss-prot), and the GO terms of differentially enriched genes were determined using hypergeometric distribution. To determine the signaling pathways related to the DEGs, KEGG pathway enrichment analysis was performed using EdTBtools [66].

### Measurement of disease index

Free PRO content was measured using sulfosalicylic acid extraction and the acidic ninhydrin staining method, according to a previously described method [67]. SOD activity, H_2_O_2_ concentration, and GSH content were measured using SOD (G0101F), H_2_O_2_ (G0112F), and GSH (G0206F) assay kits purchased from Suzhou Grace Biotechnology (Suzhou, China). The CAT content was determined using a UV spectrophotometric method [68]. MDA content was quantified through a thiobarbituric acid method [69]. Hydroxyl radicals were quantified using the plant hydroxyl radical (OH-)ELISA kit (Yaji, China, 7440-60-0). Free JA and SA were analyzed by high-performance liquid chromatography-mass spectrometry (HPLC–MS/MS) [70]. Each experiment was repeated five times. GraphPad Prism 8.0 was used for statistical and graphical analyses. The disease index (*D*) was calculated as 100 × ∑ (number of leaf discs at all levels × relative grade value)/(total number of leaf discs × highest-grade value).

Disease susceptibility was defined by the *D* scores: high resistance (HR), *D* < 10; resistance (R), 10 ≤ *D* < 25; tolerance (T), 25 ≤ *D* < 40; susceptibility (S): 40 ≤ *D* < 65; and high susceptibility (HS): *D* ≥ 65.

### Weighted gene co-expression network analysis

WGCNA was performed with the WGCNA R package (v1.70, https://cran.r-project.org/web/packages/WGCNA/index.html). A standardized gene expression matrix was used as the input. After calculating the variation in the expression of each gene, 30 344 DEGs with the most marked variation were selected for WGCNA. After threshold screening, β = 16 was chosen as the suitable value to perform power processing on the original scale-dependent matrix to obtain a scale-free adjacency matrix. To better evaluate the correlation between gene expression patterns, the adjacency matrix was further transformed into a topological overlap matrix (TOM), and the topological dissimilarity matrix (dissTOM = 1 − TOM) was used for gene clustering and module division using the dynamic shear algorithm. The minimum number of genes in the module was 30 (minModuleSize = 30), the threshold for merging similar modules was 0.25 (cutHeight = 0.25), and the network type was unsigned. To identify modules specific for disease resistance, the correlation coefficient *r* and corresponding *P* values between the module feature vectors of each module and different disease resistance traits were calculated. Modules with a positive correlation (*r* > 0) and high significance (*P* < 0.01) were selected for further analysis. The network was visualized using Cytoscape v3.6.1.

### Transient overexpression in kiwifruit leaf and quantitative RT–PCR analysis

The total RNA samples used for RNA-seq were subjected to qRT–PCR. Two micrograms of total RNA was used for reverse transcription, and first-strand cDNA was synthesized using a RevertAid First Strand cDNA Synthesis Kit (Thermo Fisher Scientific, K1622). qPCR was conducted using the Chamo Universal SYBR qPCR Master Mix (Vazyme Biotech, China) and run on a LightCycler 480 II quantitative instrument (Roche, Switzerland), according to the manufacturer’s instructions. Kiwifruit β-actin (GenBank ID ABR45727.1) was used as the reference gene, and relative gene expression was measured by the 2^−ΔΔCt^ method. Primers used for qPCR are listed in Supplementary Data Table S9.

This study was based on a previous study [71] and we selected leaves of the susceptible kiwifruit *A. chinensis* var. *chinensis* ‘Hort16A’ with the same growth status. Next, the fusion gene carrying *Agrobacterium* GV3101 was overexpressed in kiwifruit leaf discs using the vacuum infiltration method. Leaf discs were placed on 0.7% water agar and cultured in a light incubator (16 h light/8 h dark, 20°C). After 48 h of culture, the samples were subjected to qRT–PCR for gene expression analysis. The leaf discs were inoculated with *Psa* (M228) using the vacuum infiltration method and placed in an incubator (16°C). After 5 days, photographs were taken to observe the phenotype, and ImageJ (V1.8.0) was used to calculate the disease index. The number of bacteria colonizing the leaf discs was counted using the spread-plate method. Graphpad Prism (V9.2) was used for data visualization [71]. The experiments were independently replicated at least three times, and the data are presented as the mean ± standard deviation of three independent experiments. The fusion expression vectors used to construct primers are listed in Supplementary Data Table S10.

## Supplementary Material

Web_Material_uhad242

## Data Availability

The raw RNA-Seqdata that support the findings of this study are openly available in NCBI at [https://www.ncbinlm.nih.gov/sra/PRINA1054973], reference number [PRJNA1054973].
