# Supplementary material for: Two transcription factors, AcREM14 and AcC3H1, enhance the resistance of kiwifruit Actinidiachinensis var. chinensis to Pseudomonas syringae pv. actinidiae
Source: Hortic Res. 2023 Nov 20;11(1):uhad242. doi: 10.1093/hr/uhad242 (PMC10782502; doi:10.1093/hr/uhad242)
Supplement: Web_Material_uhad242 [file web_material_uhad242.zip › Suplement Figure and Legend.docx]

**Figure legend:**

**Fig. S1**. Kiwifruit leaf disc assay evaluating *Psa*-susceptibility of the F1 offspring. *A. chinensis* var. *deliciosa* ‘Xuxiang’ (XX, resistance), *A. chinensis* var. *deliciosa* ‘Cuixiang’ (CX, resistance)., *A. chinensis* var. *chinensis* ‘Hongyang’ (HY, High susceptible), *A. chinensis* var. *deliciosa* ‘Hayward’ (HWD, Tolerance) and *A. macrosperma* var. ‘Dazi’ (DZ, High resistance) are the five controls. Letters indicate statistical significance (Duncan's multiple range test，*P*＜0.05; different letters denote significant).

**Fig. S2**. Detection of plant defense-related molecules in *A. chinensis* var. *chinensis* hybrids RH12 and SH14 following *Psa* infection. (a)-(i) line charts demonstrating the changes in H_2_O_2_, MDA (malondialdehyde), electrolyte leakage (%), OH hydrogen peroxide levels, PRO (proline), GSH (glutataione), CAT (catalase) activity, and SOD (superoxide dismutase) activity,SA (salycilic acid) and JA (Jasmonic acid) content. Hort16A and Xiong22 are used as positive and negative controls, respectively. Xiong22, *A. chinensis* var. *chinensis* ‘Xiong22’; Hort16A, *A. chinensis* var. *chinensis* ‘Hort16A’. Data are presented as means ± SD, each assay was repeated at least three times. Letters above the error bars indicate statistical significance (Fisher’s protected LSD test, *p* < 0.05, different letter indicate statistically significant). FW, fresh weight.

**Fig. S3.** PCA(principal component analysis) examining sample variation.

**Fig. S4.** GO enrichment of (a) profile2 (SH14) and (b) profile17 (RH12) genes. The red marks indicate the Go terms that are present in both profile2 and profile17. In addition, the heatmaps (c-h) represent the expression of differentially expressed genes (DEGs) associated with the following six Go terms: response to external stimulus (GO:0009605), plasma membrane (GO:0005886), vacuole (GO:0005773), cell wall (GO:0005618), external encapsulating structure (GO:0030312), and transferase activity (GO:0016740). The colors ranging from green to red indicate the expression of genes from low to high, respectively.

**Fig.S5.** KEGG pathway analysis for the ten comparison groups. The size of the circle indicates the level of Gene Ratio. From blue to red indicates the change in *p*-value from small to large.

**Fig.S6.** The three pathway genes enriched by KEGG were analyzed in detail. (a) Profiles of differentially expressed genes (DEGs) mapped to the hormone signaling pathway. (b) Profiles of DEGs mapped to the pathways involved in plant-pathogen interactions. (c) Profiles of DEGs mapped to the flavonoid biosynthesis-related pathway. See Table S3 for details on the genes shown in the heatmap. Colors from green to red indicate gene expression from low to high, respectively.

**Fig.S7.** Determination of soft-thresholding power (β) for the WGCNA analysis. (a) Scale-free topology fit index as a function of the soft-thresholding power. The red line indicates that R^2^ is equal to 0.85. (b) Mean connectivity as a function of the soft-thresholding power. (c) Correlation analysis among the WGCNA modules.

**Fig.S8.** Construction of weighted gene co-expression network analyses (WGCNA) and module detection. (a) Hierarchical cluster diagram showing co-expression modules according to WGCNA. (b) Heatmap analysis of correlations between samples and traits. (c) Heatmap analysis of correlations between modules and traits. Module names in red are the modules associated with height and character. Each cell contains the corresponding correlation and p-value. RS, resistance and susceptibility. ME, module eigengenes.

**Fig.S9**. Correlation of traits and modules. Scatterplots showing gene significance versus module membership for the MEgreen/H2O2 (a), MEturquoise/ELT (b), MEblack/GSH (c), MEpink/R.S (d), with correlations and indicated p-values.

**Fig.S10**. Expression patterns, function enrichment, and correlation networks of MEgreen and MEblack modules. (a) and (b), eigengene expression profiles for the MEgreen and MEblack modules. The y-axis indicates the eigengene values in the module; the x-axis shows samples at different infection time points. (c) and (d), GO term enrichment of the MEgreen and MEblack module genes. Non-redundant GO terms are visualized by the TreeMap view of REVIGO. Rectangle sizes reflect adjusted p-values (*p*<0.05). (e) and (f), Correlation networks of the MEgreen and MEblack modules. Top 5 % of genes with the highest degree (most edges) in each module were selected as hub genes. Arrowheads represent TFs.

**Fig.S11.** Expression patterns, function enrichment, and correlation networks of MEpink and MEturquoise modules. (a) and (b), Eigengene expression profiles for the MEpink and MEturquoise modules. The y-axis indicates eigengene values in the module. The x-axis shows samples at different infection time points. (c) and (d) GO term enrichment of genes from MEpink and MEturquoise modules. Non-redundant GO terms are visualized by the TreeMapview of REVIGO. Block sizes reflect the adjusted p-values (*p*<0.05). (e) and (f) correlation networks of genes from the MEpink and MEturquoise modules. Top 5 % of genes with the highest degree (most edges) in each module were selected as hub genes. Arrowheads represent TFs.

**Fig.S12**. Quantitative RT-PCR verification of selected hub genes. Bar and line charts indicate the gene expression data from RNA-seq and qRT-PCR. Red and blue bars denote the expression results derived from RH12 (R) and SH14 (S).

**Fig.S13**. Identification of overexpression status and disease incidence in leaves after gene overexpression.(a) qRT-PCR analysis of the expression of the three genes in kiwifruit leaf discs. (b) Disease incidence of the leaf discs.PC, the positive control, refers to the transient overexpression of the empty vector pCABIA1302 in Xiong22, followed by inoculation with Psa. Similarly, the transient overexpression of the empty vector pCAMBIA1302 in kiwifruit Hort16A leaves, followed by inoculation with Psa, serves as the negative control(NC).

**Fig.S14**. Multiple sequence alignment analysis of homologous proteins of AcREM14 in kiwifruit and other species.

**Fig.S15**. Multiple sequence alignment analysis of homologous proteins of AcC3H1 in kiwifruit and other species.

**Fig.S16**. Clone and align the promoter regions of AcREM14 and AcC3H1. a and b represent the gene AcREM14 and AcC3H1 promoter sequence alignment analysis in SH14 and RH12, respectively. Red boxes indicate cis-acting elements in the promoter region. 'ATG' represents the transcription start site.

**TableS1.** RNA-seq data metrics.

**TableS2.** Top 5 DEGs in different comparison groups.

**TableS3.** Details of DEGs mapped to the hormone pathway (a), flavonoid synthetic pathways (b), and plant-pathogens interaction pathways (c).

**TableS4.** GO term enrichment analysis of the green (a), black (b), pink (c), and turquoise (d) modules.

**TableS5.** Numbers of TFs identified in the green, black, pink, and turquoise modules.

**TableS6.** Summary of the top 5% genes present in the green, black, pink, and turquoise modules.

**TableS7.** Analysis of cis-acting elements for the six hub transcription factors.

TableS8. Interaction predictions between six hub transcription factors and top 5% hub genes.

**TableS9.** Primers used for qRT-PCR.

**TableS10.** Transient overexpression vector pCAMBIA1302 construction primers.

**TableS11.** ID names corresponding to homologous genes of AcREM14 and AcC3H1.

**Fig.S1**.


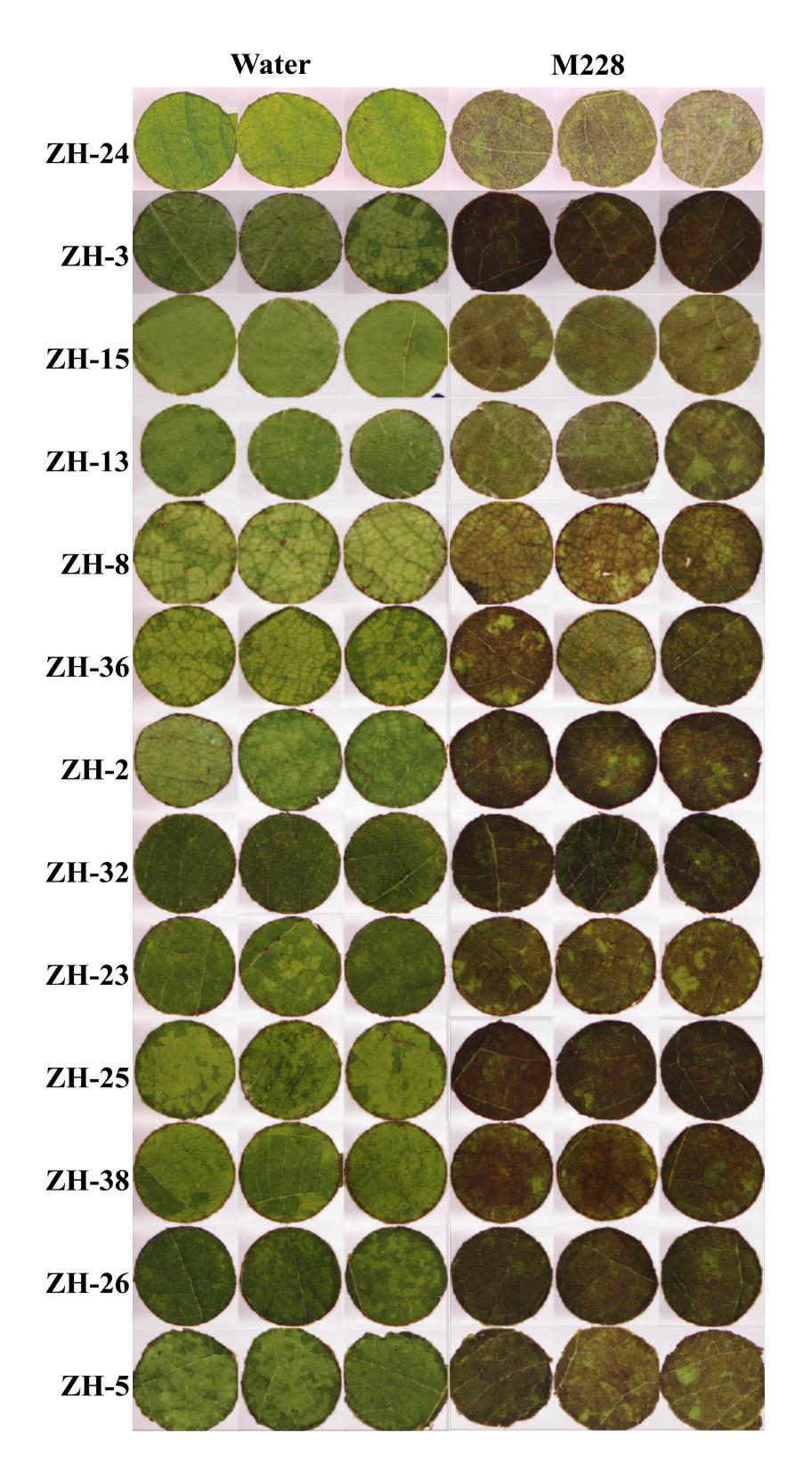

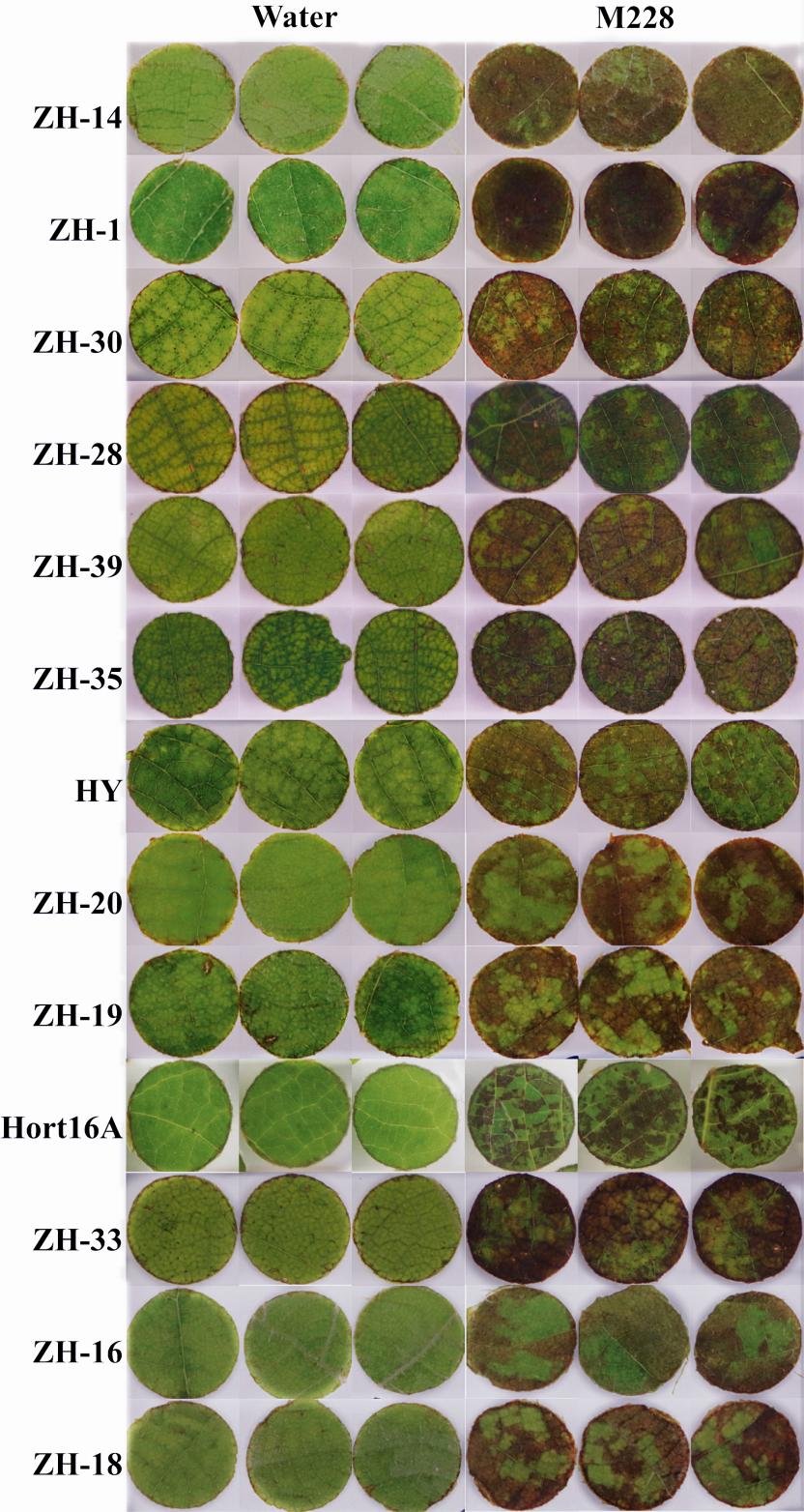


**ZH-14**

**ZH-1**

**ZH-30**

**ZH-28**

**ZH-39**

**ZH-35**

**ZH-20**

**ZH-19**

**Hort16A**

**ZH-33**

**ZH-16**

**ZH-18**

**HY**

**Water**

**M228**

**HS**

**HS**


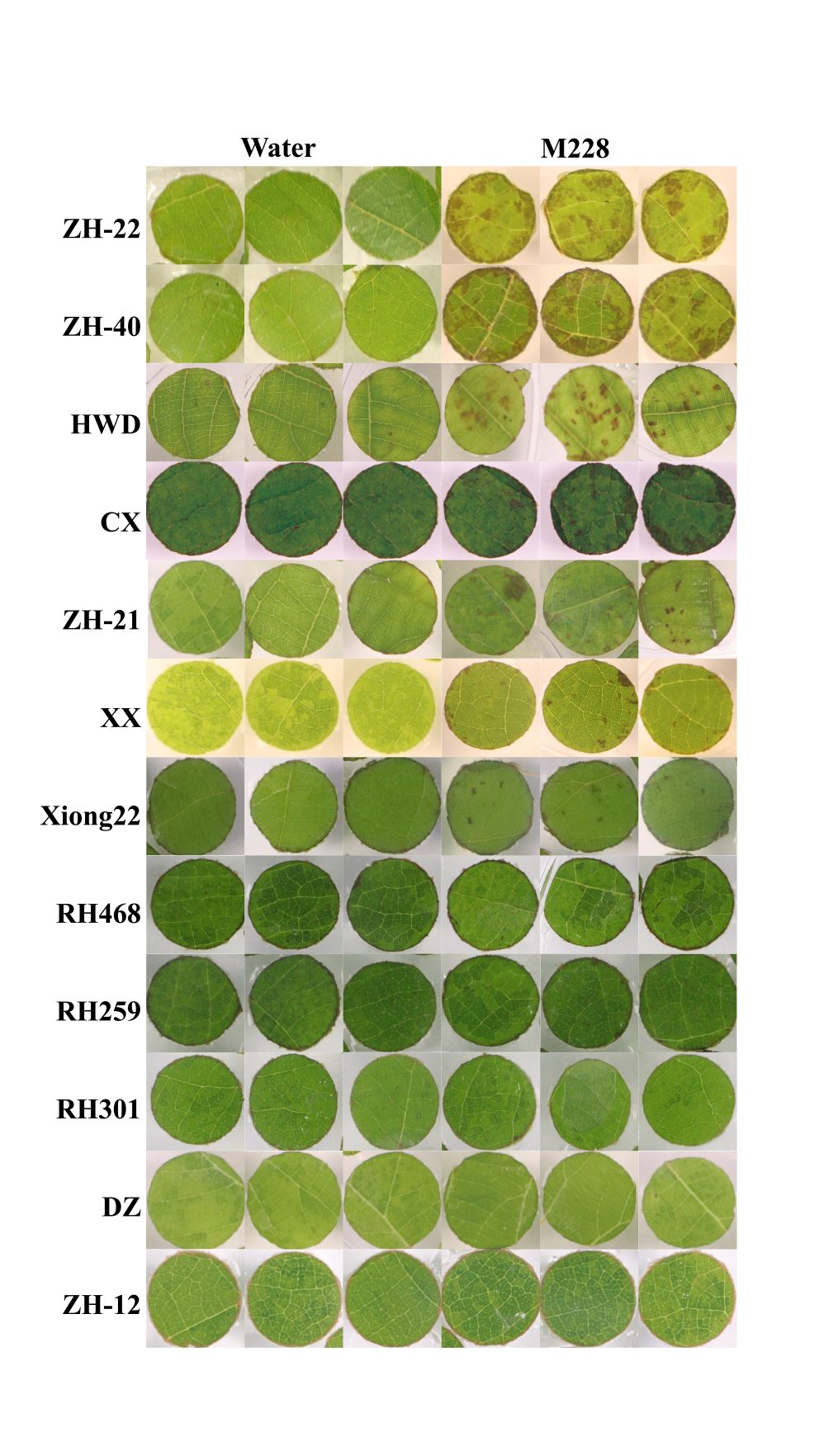


**Fig.S1**.


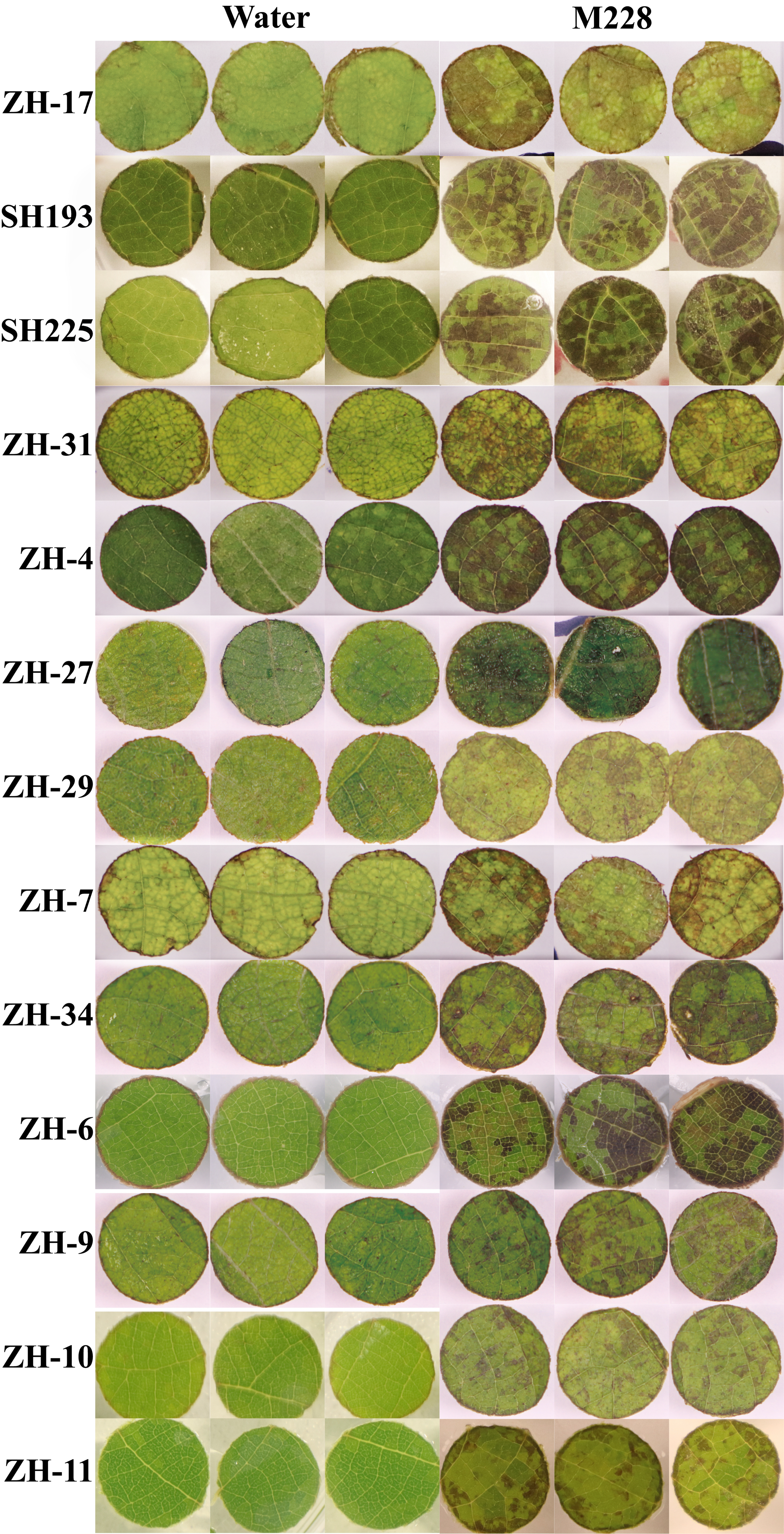

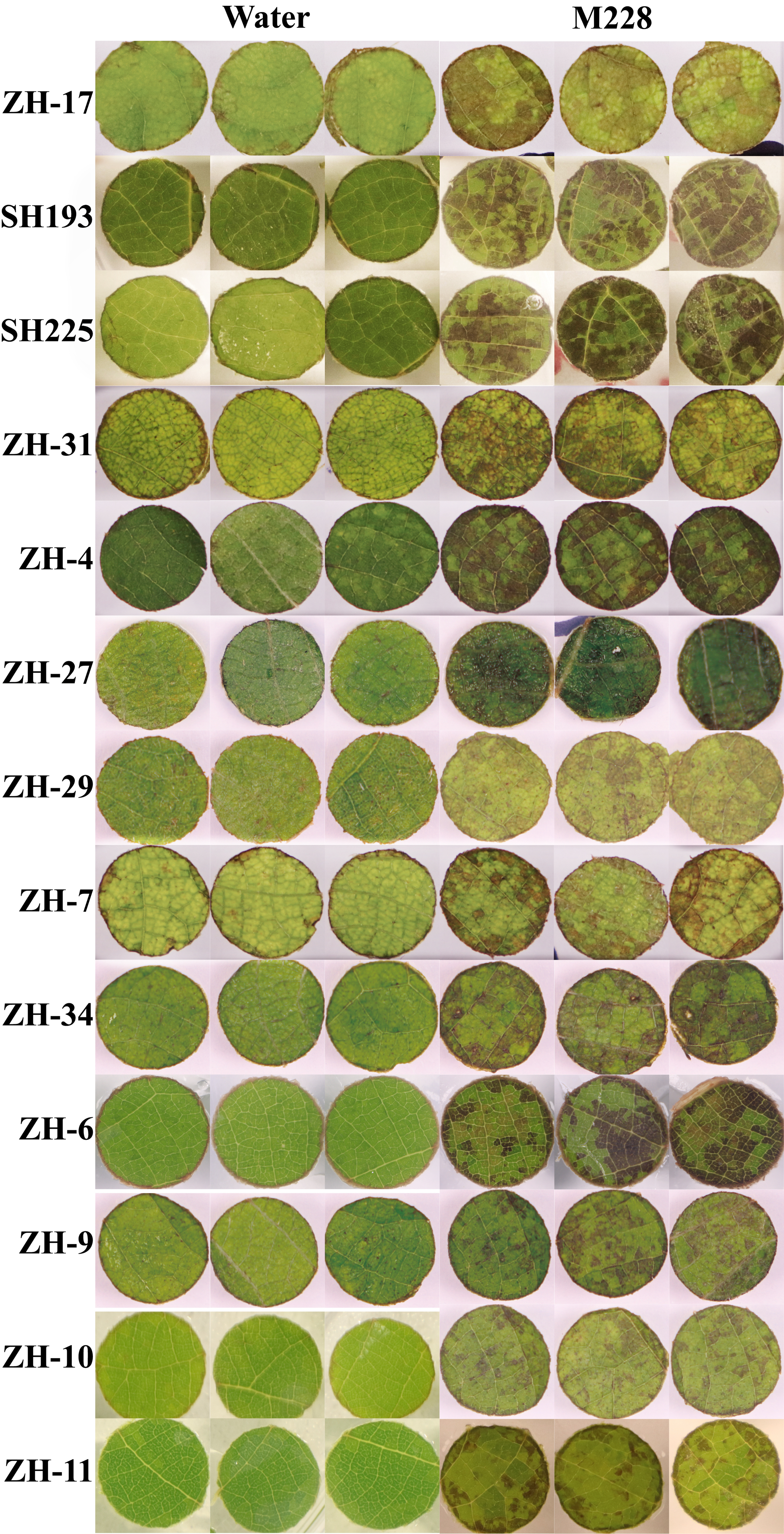

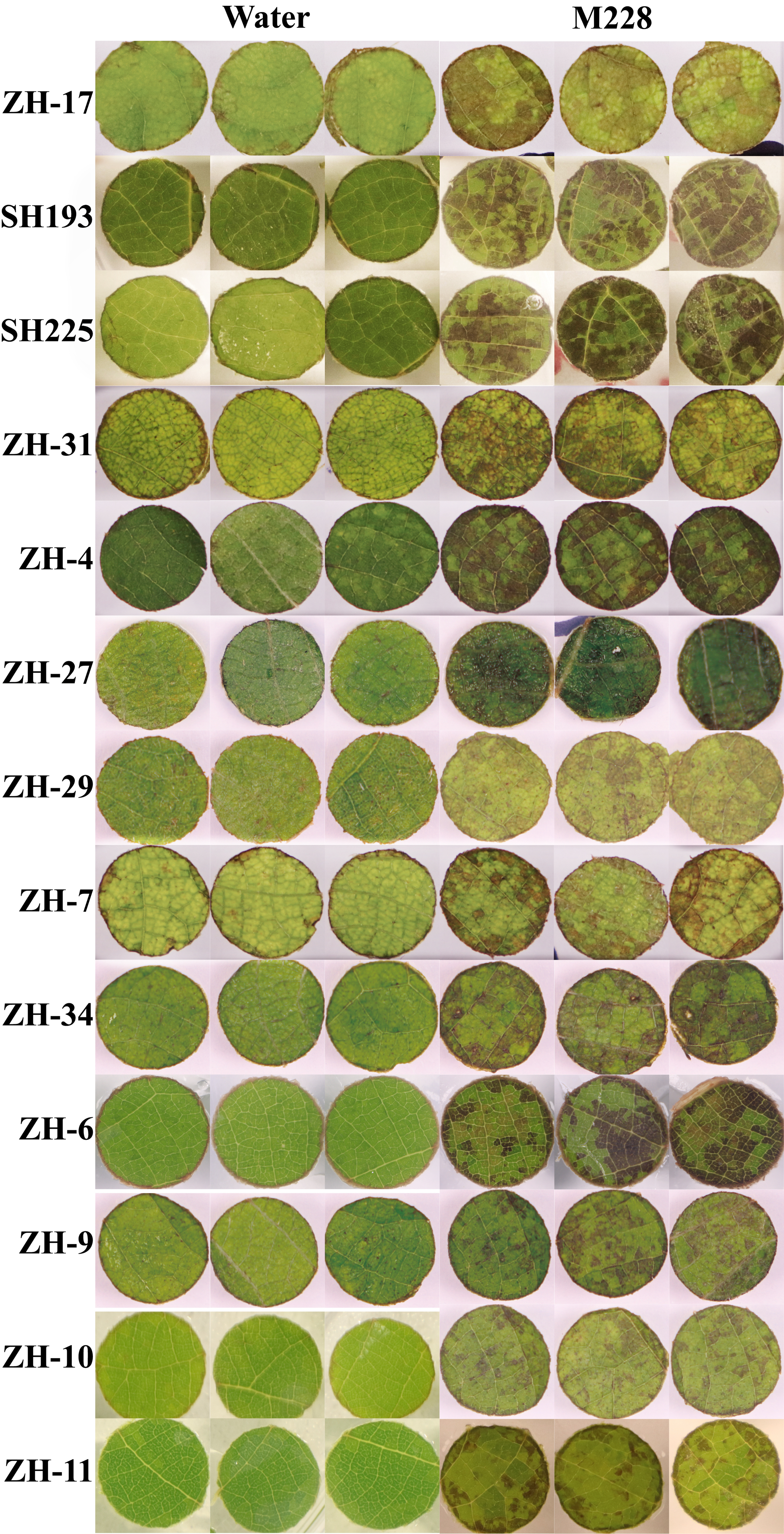


**HR**

**R**

**T**

**T**

**S**


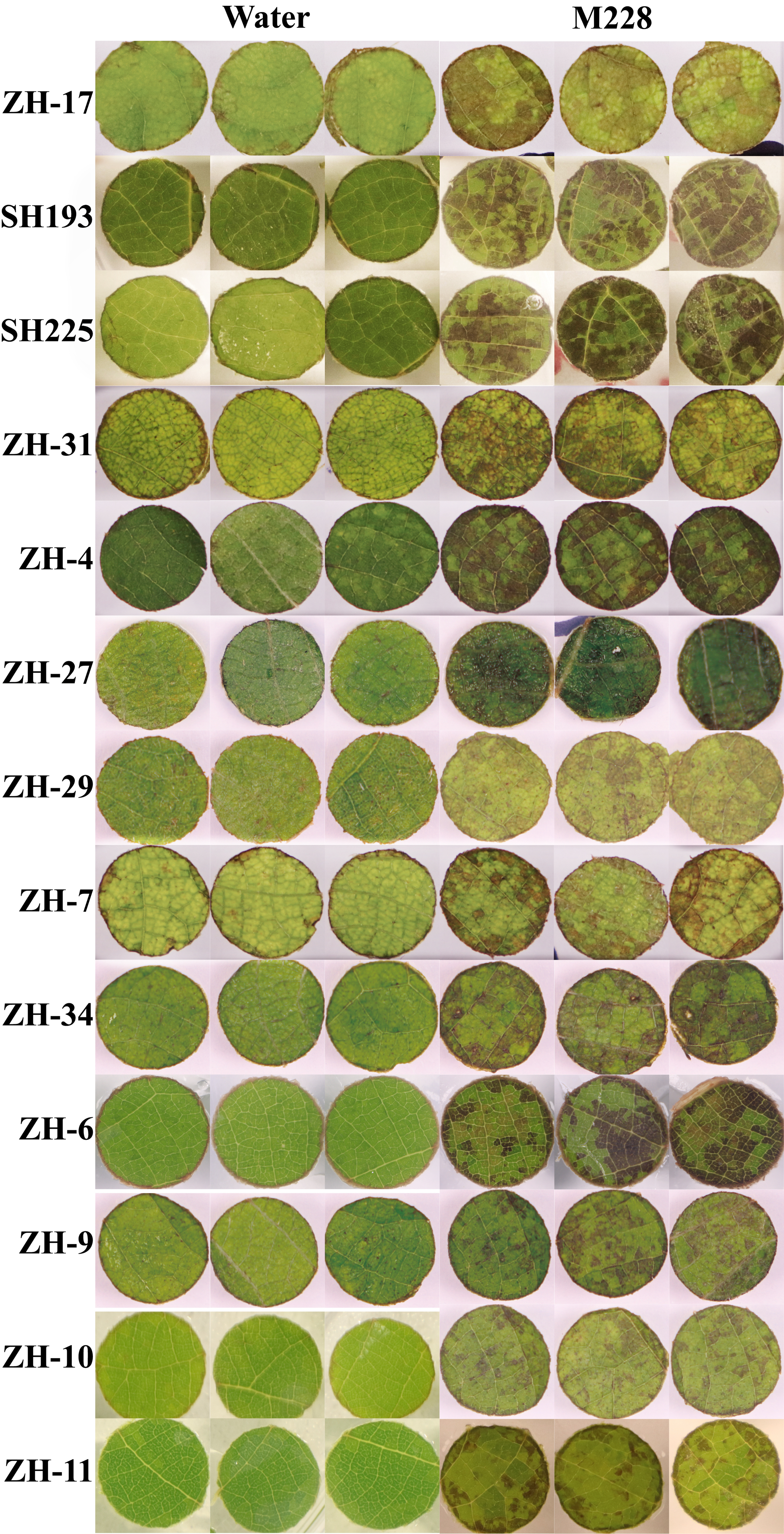

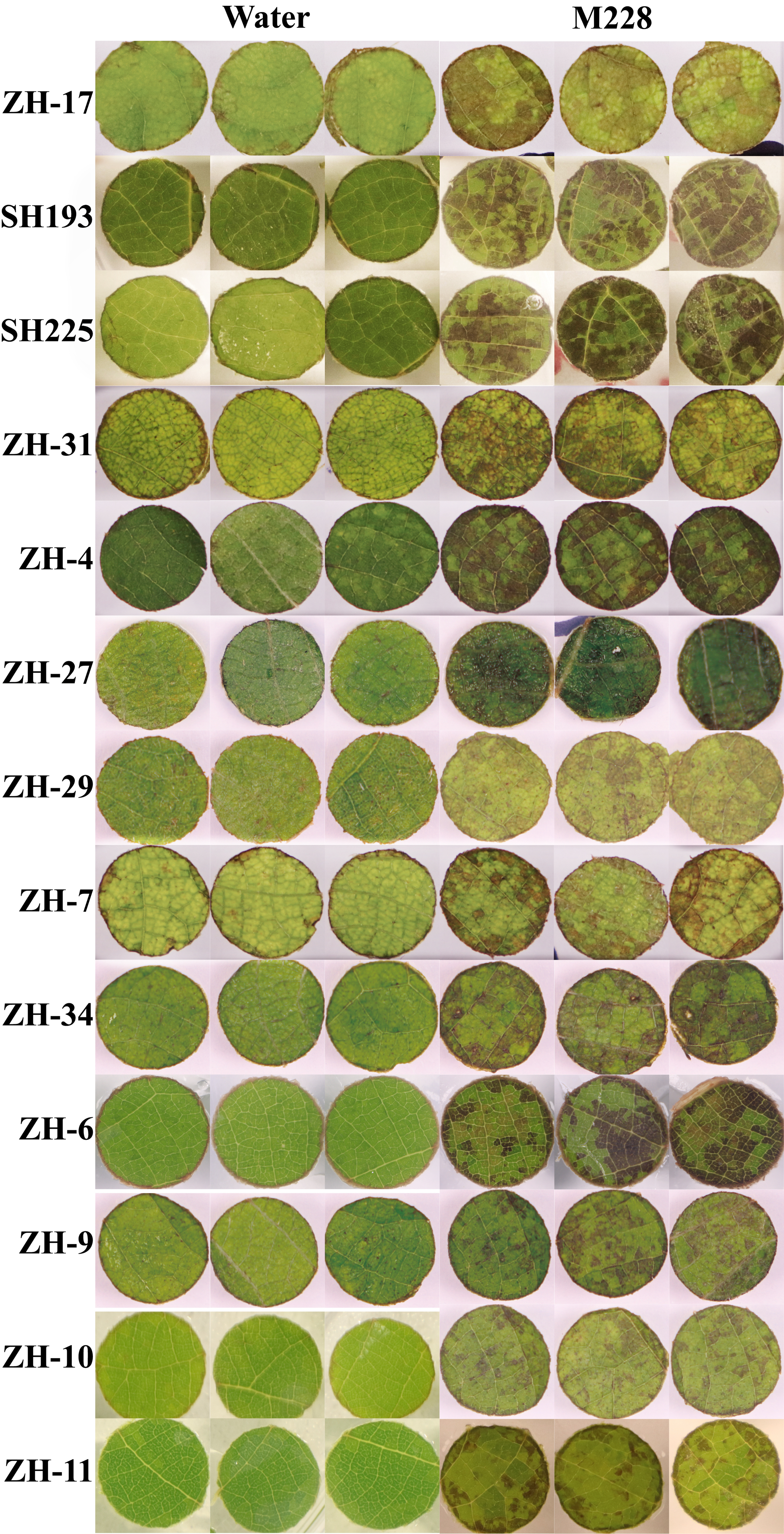


**HS**

**Fig.10.**


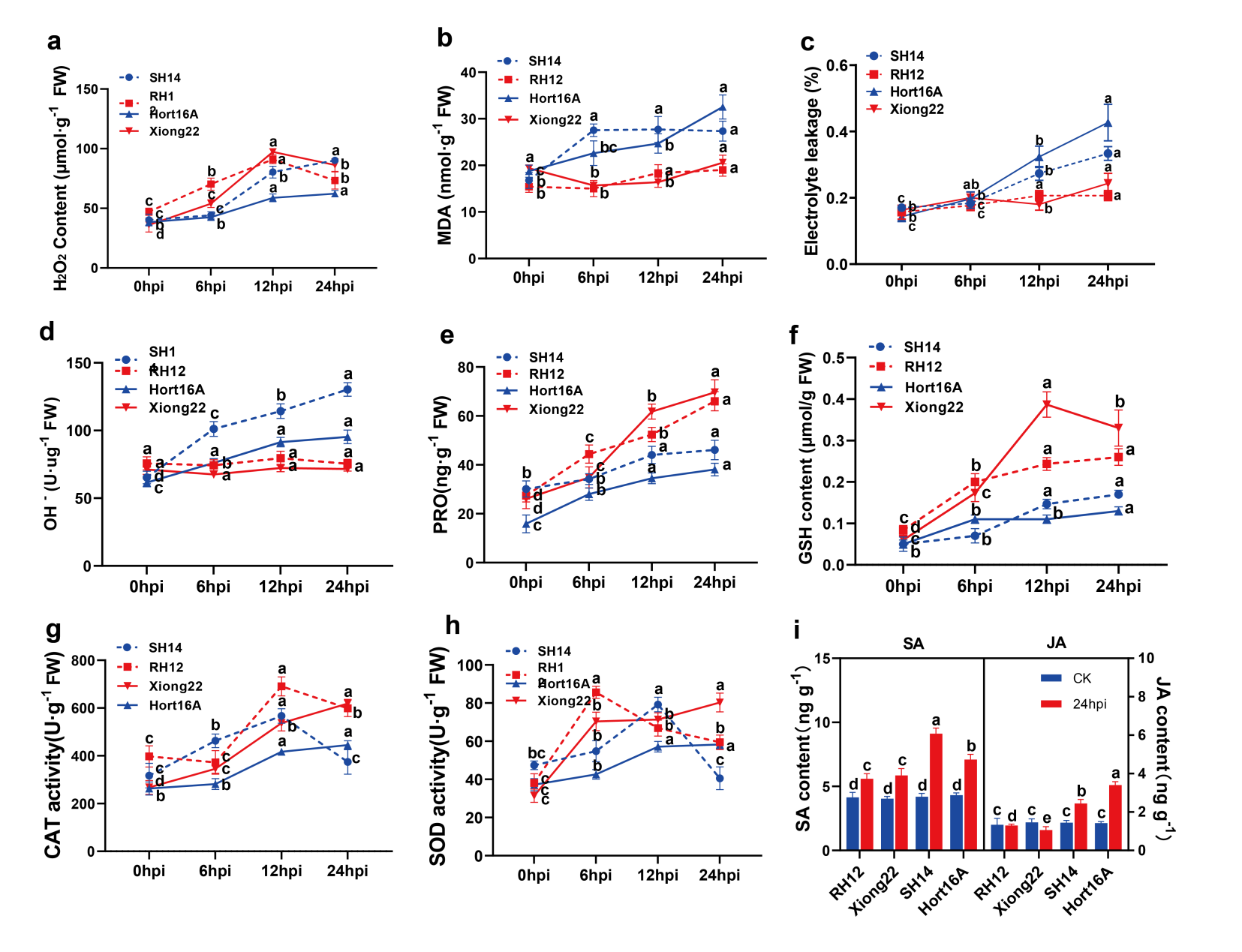


**Fig.S2.**

**Fig.S2**.

**Fig.S2**.


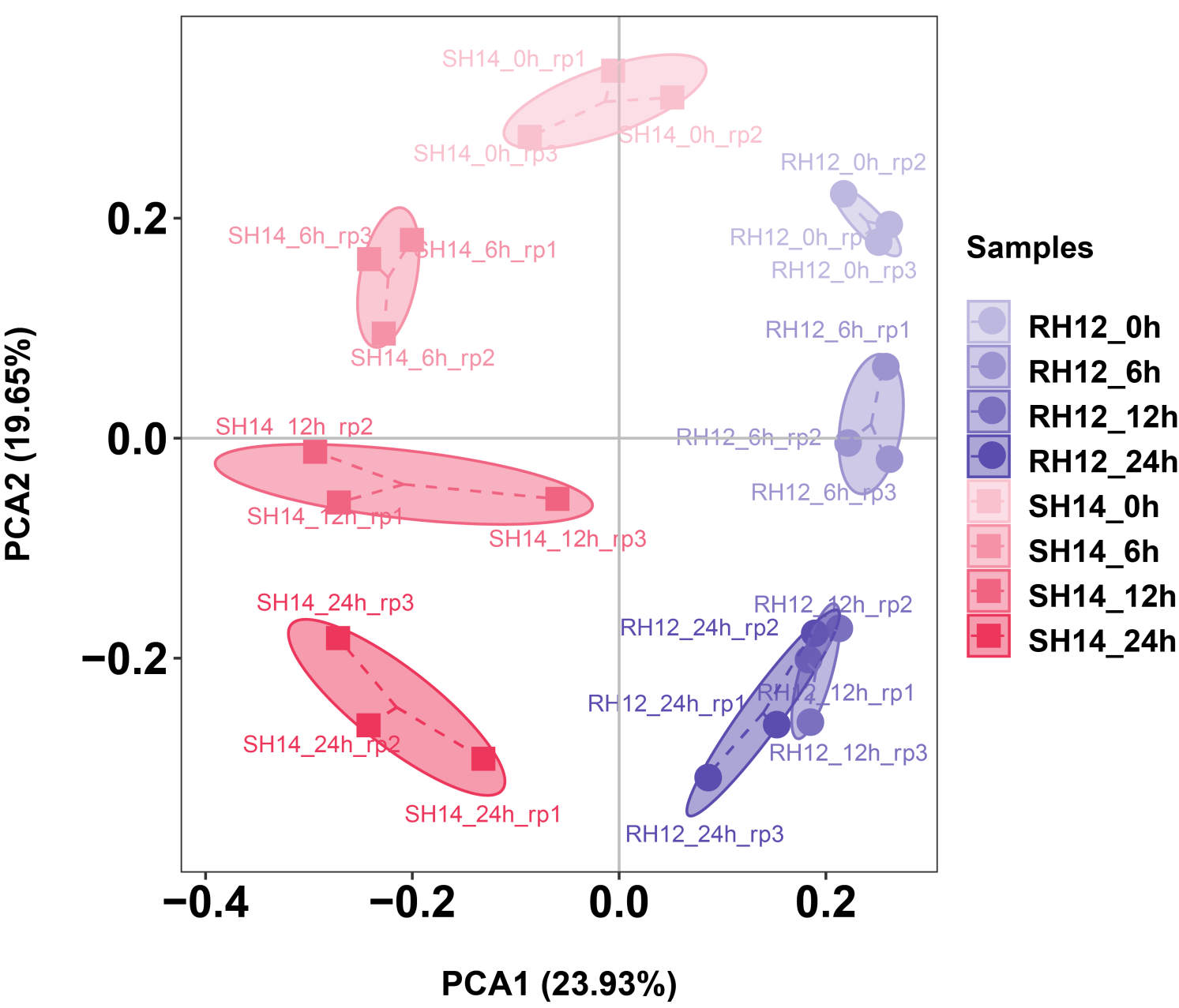


**Fig.S3.**


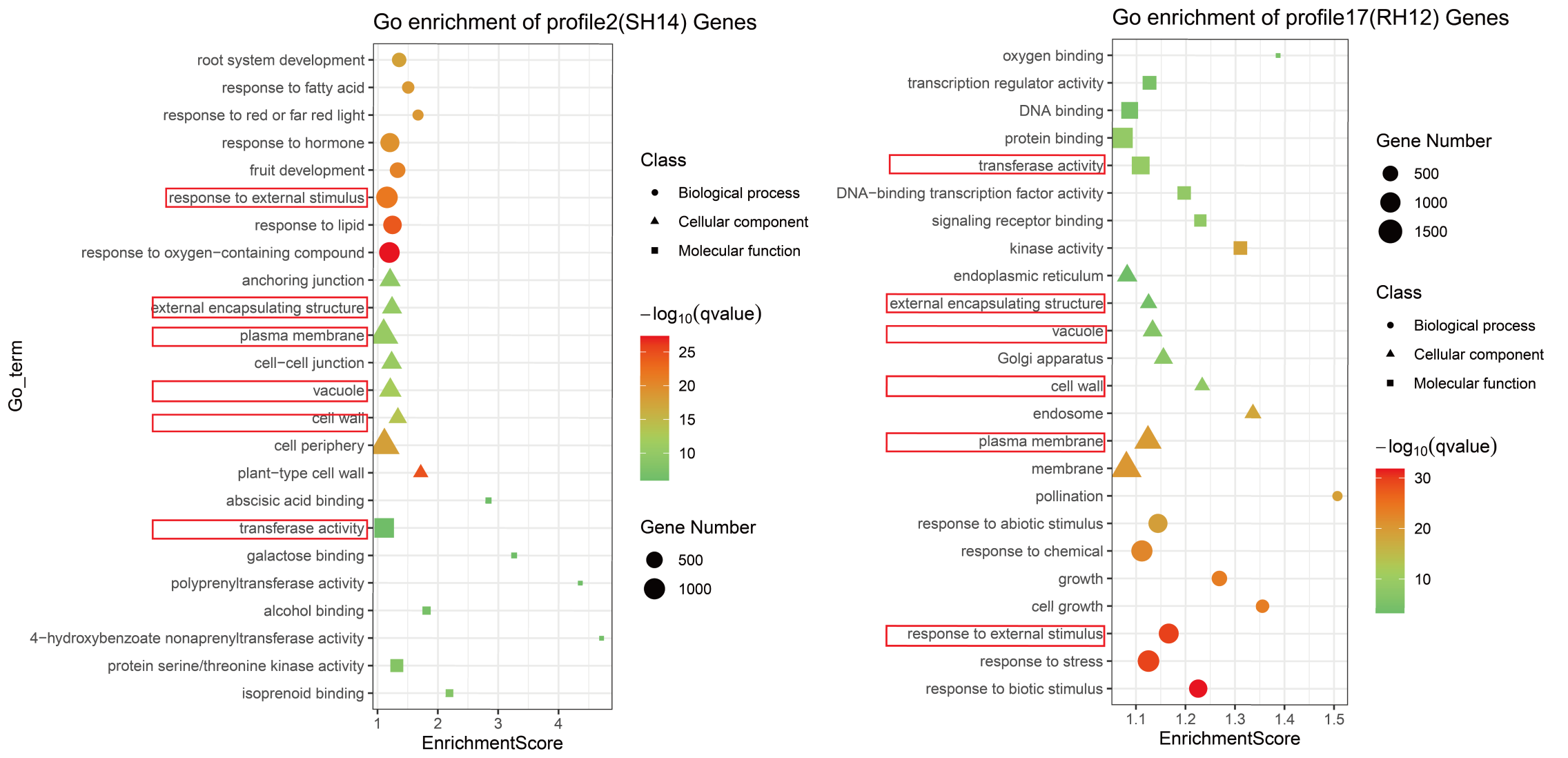


**Fig.S4.**

**b**

**a**


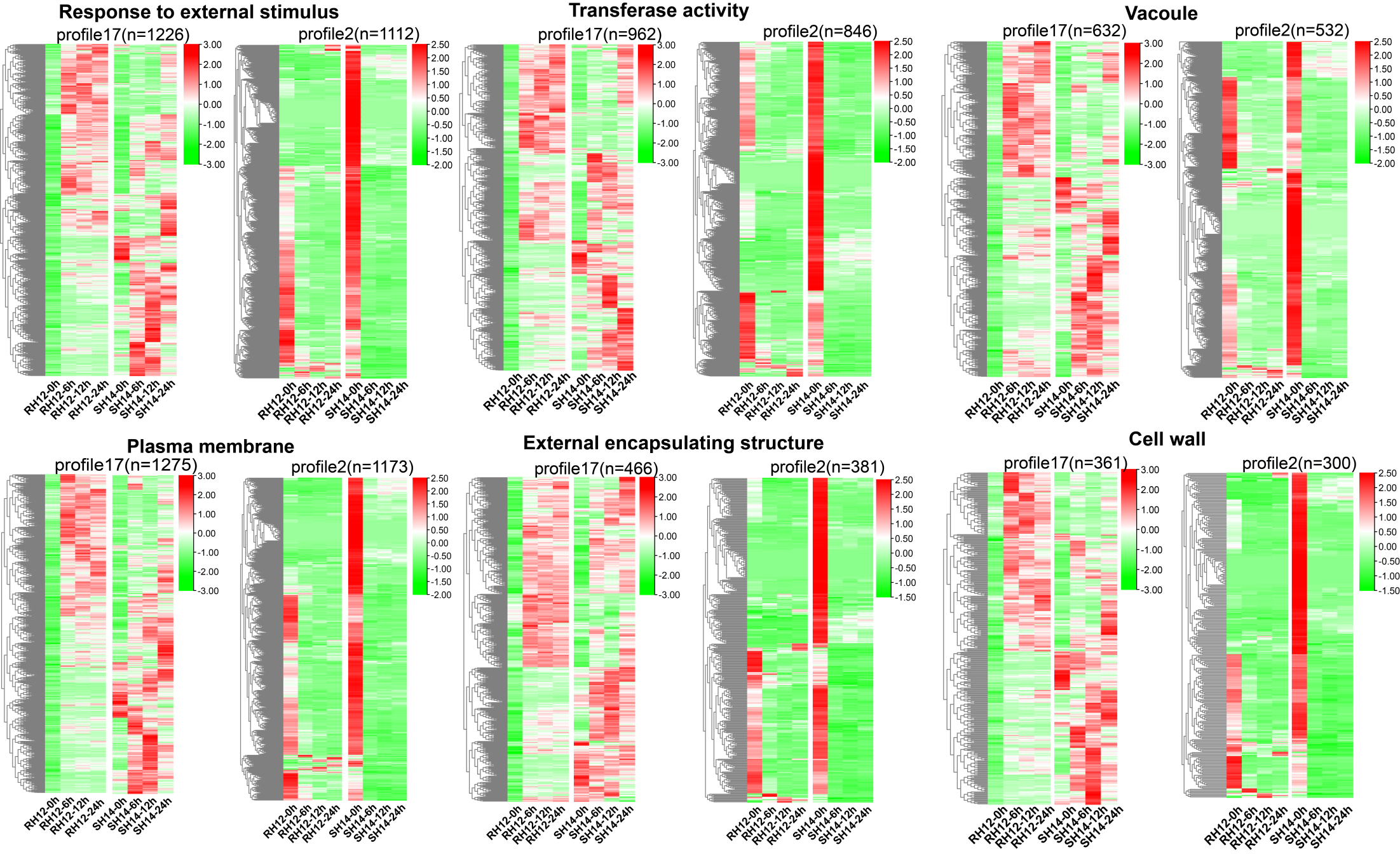


**g**

**e**

**d**

**f**

**Fig.S4.**

**h**

**c**


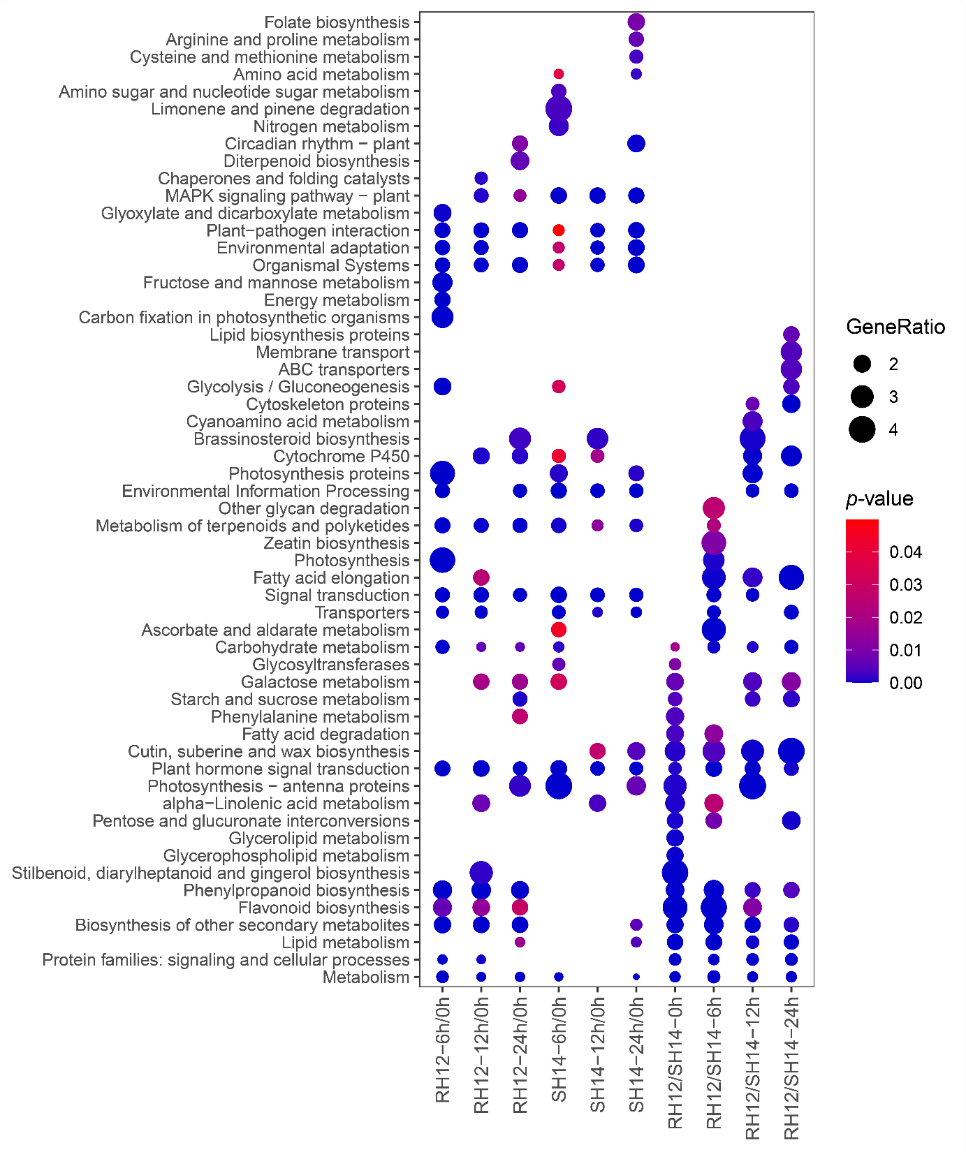


**Fig.S5.**


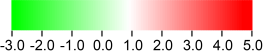

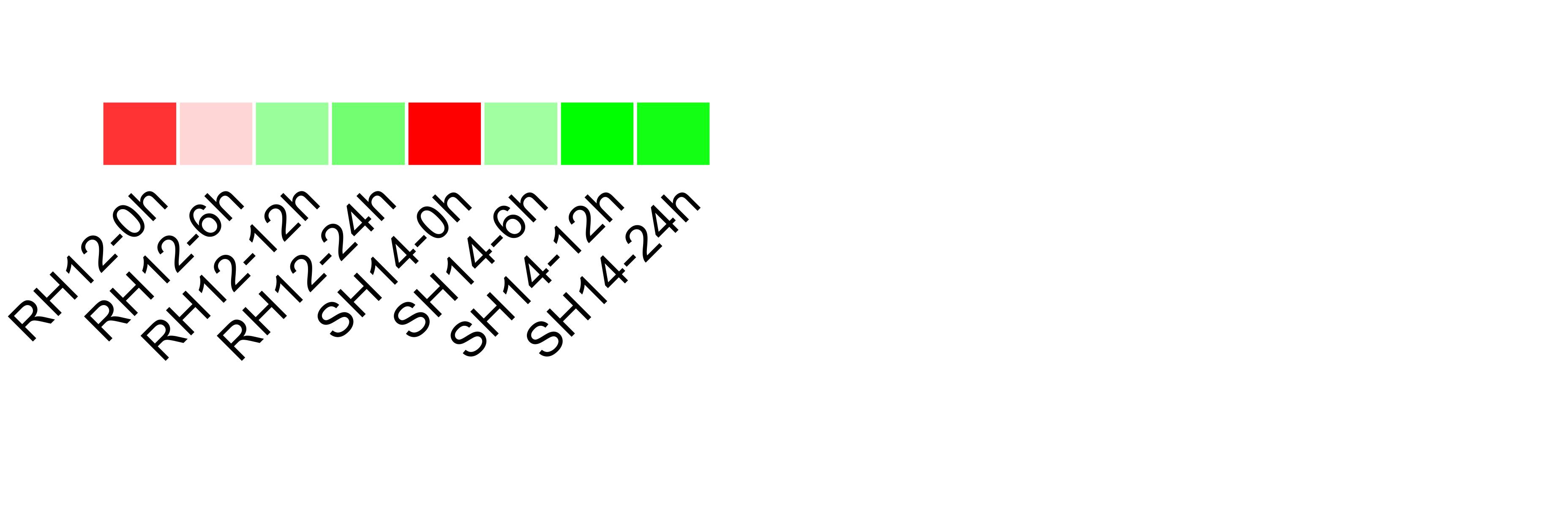

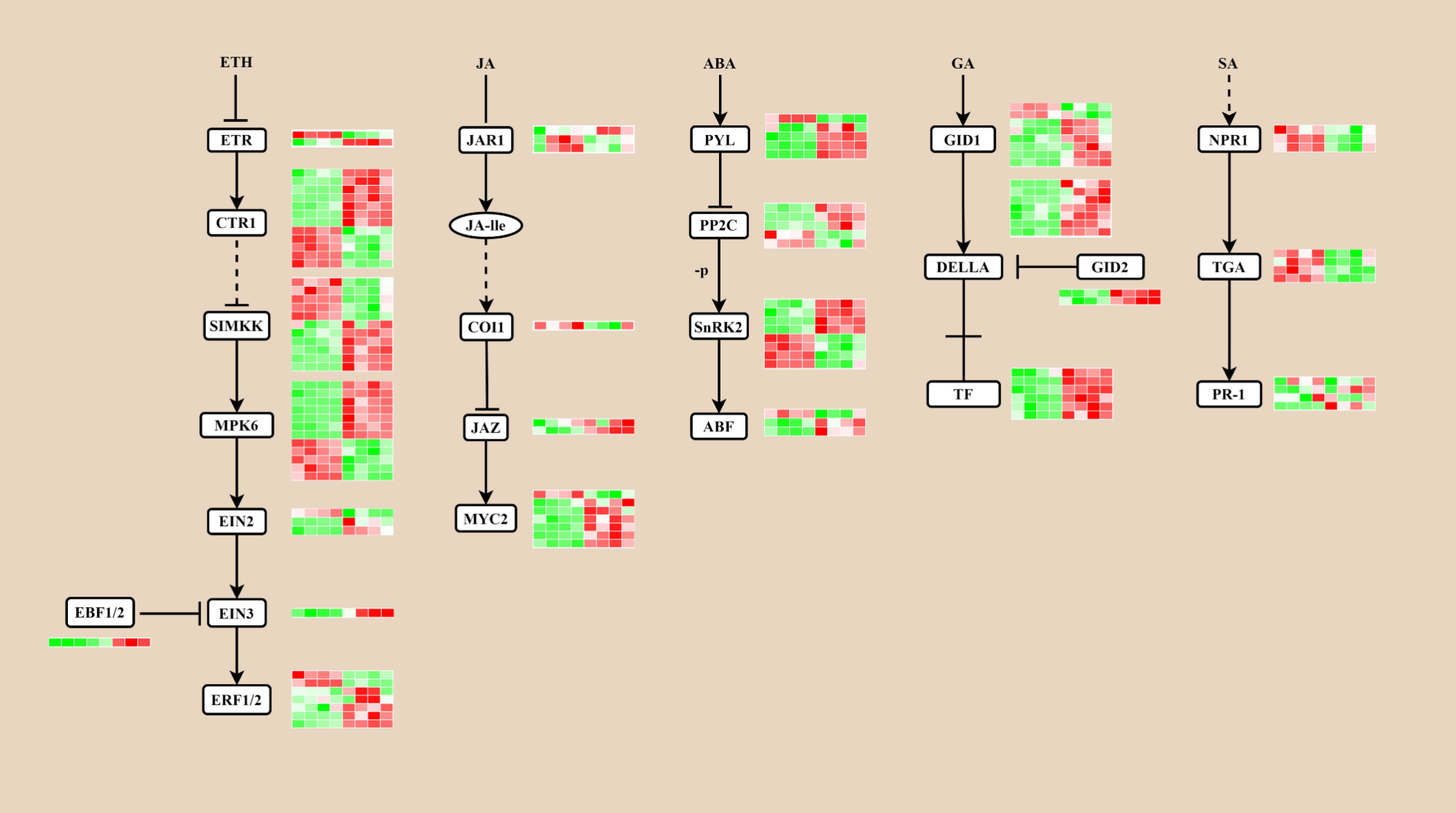


**Fig.S6. a**


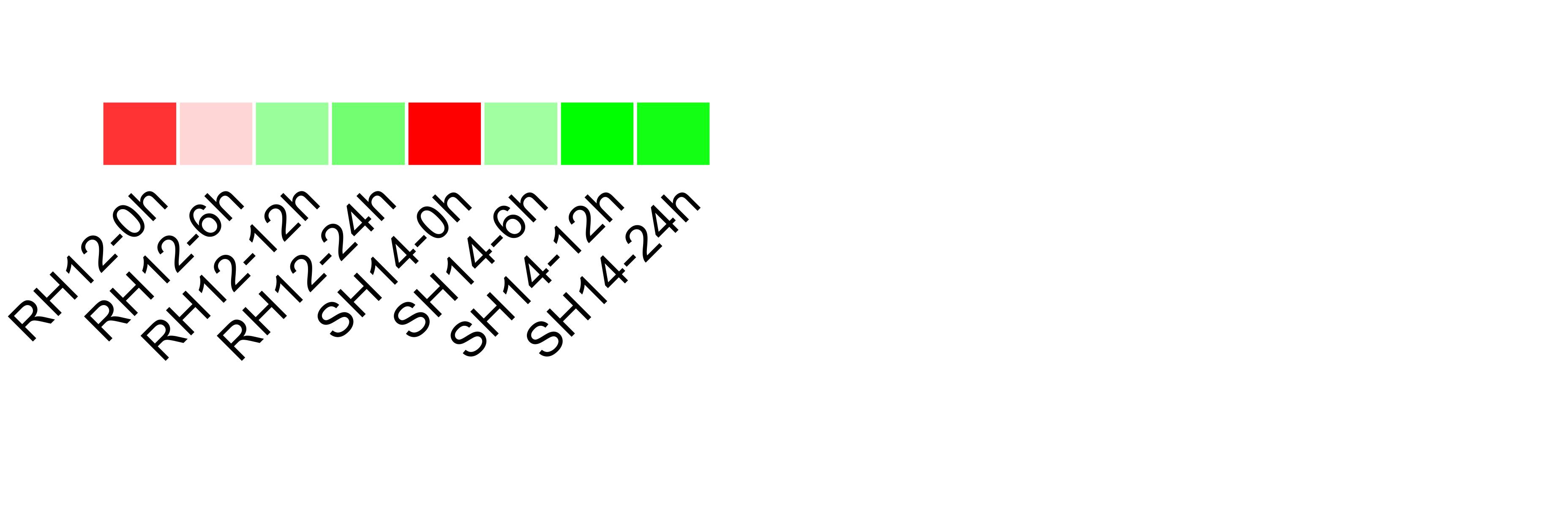

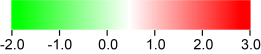

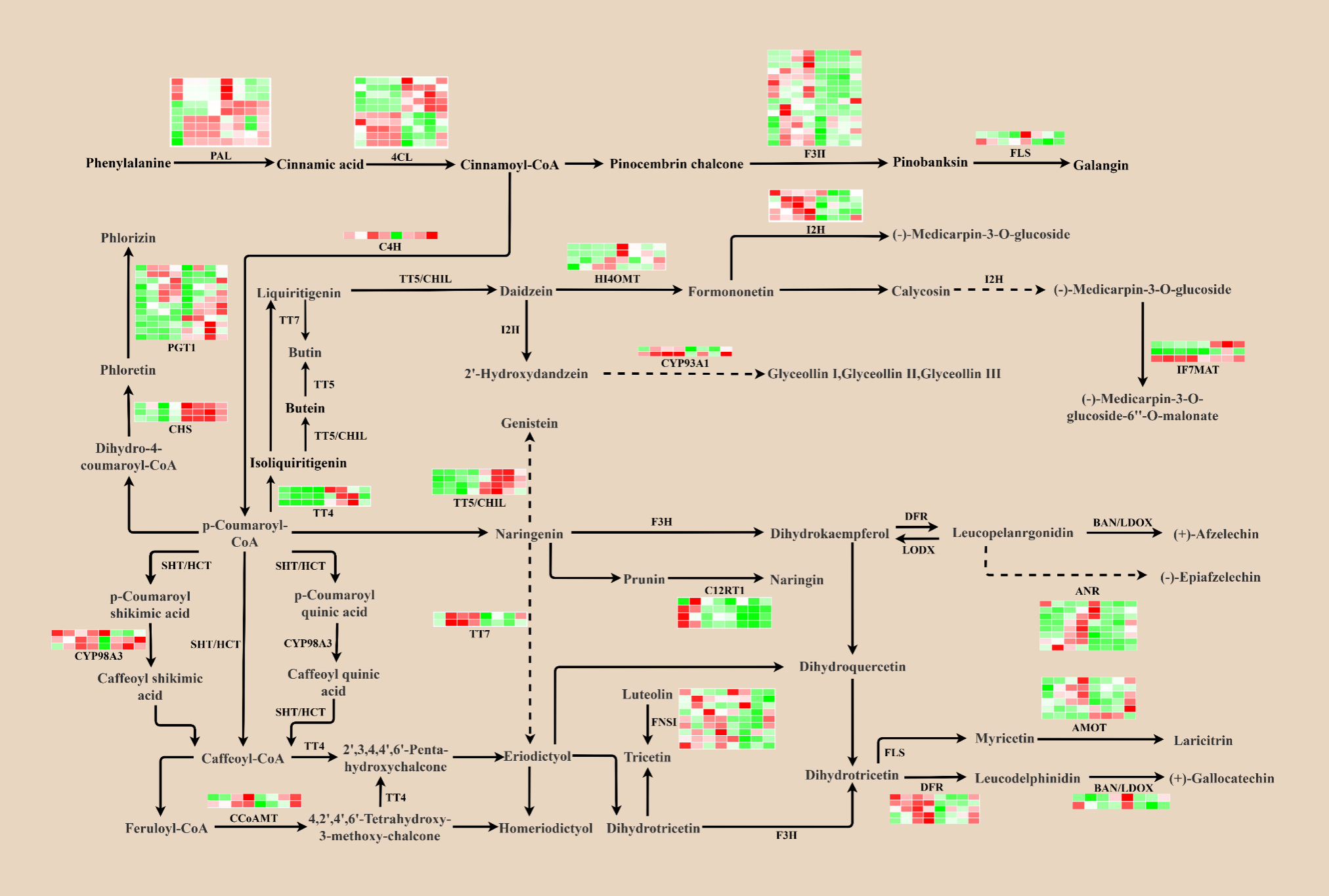


**Fig.S6. b**


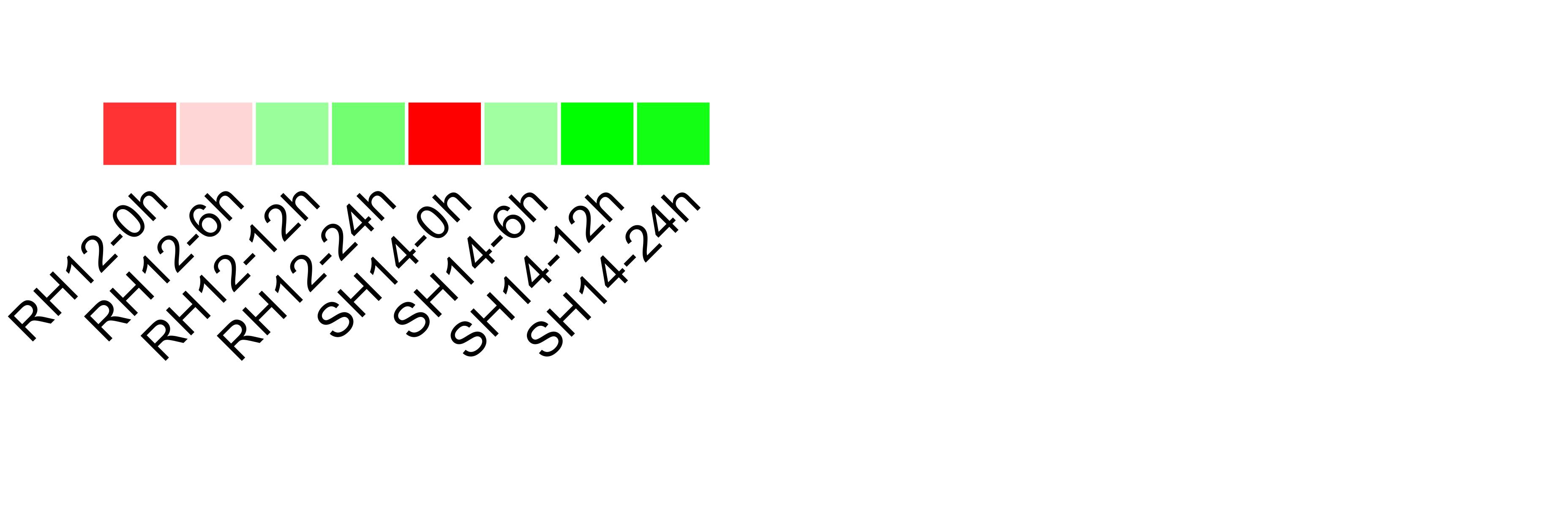

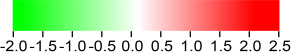

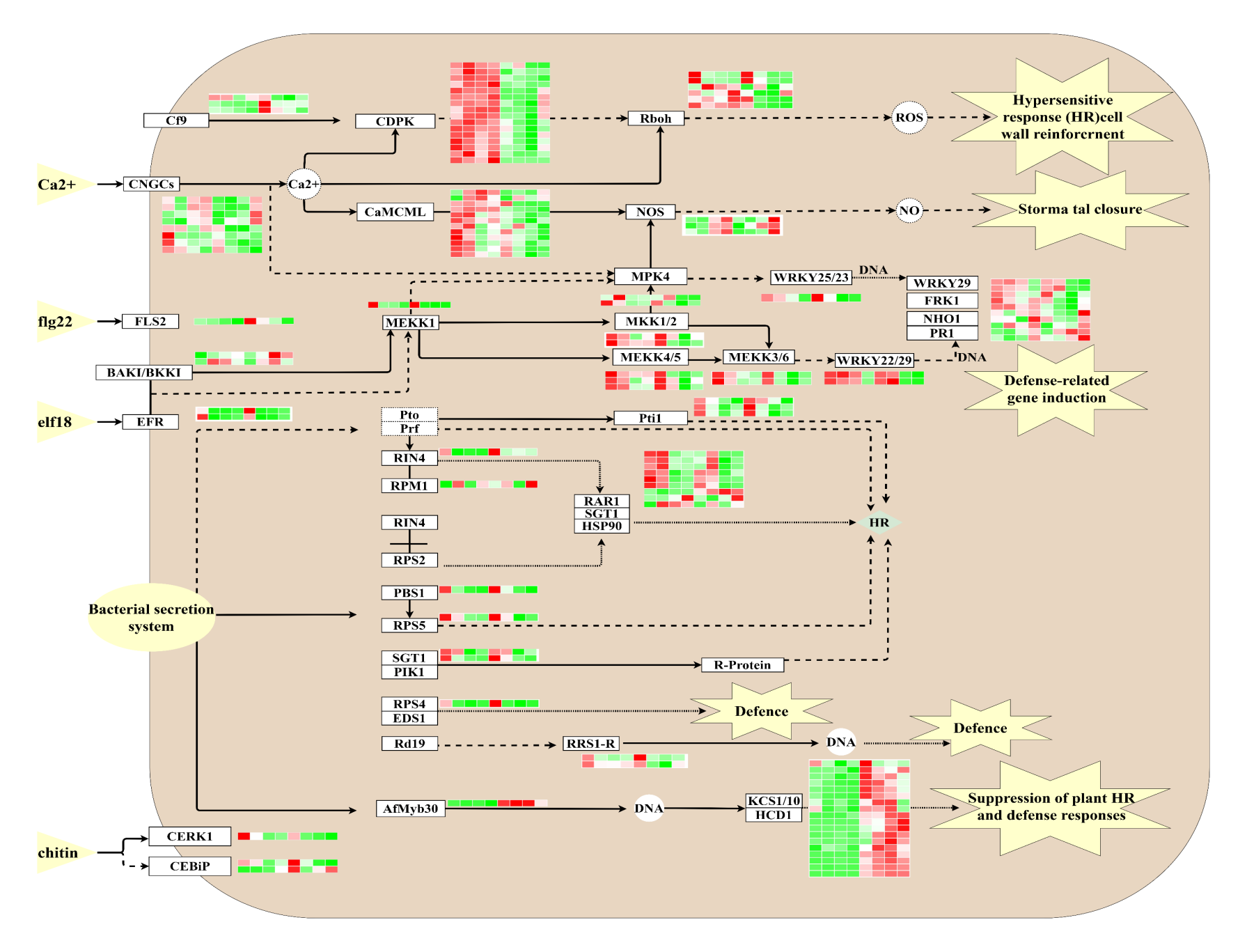


**Fig.S6. c**


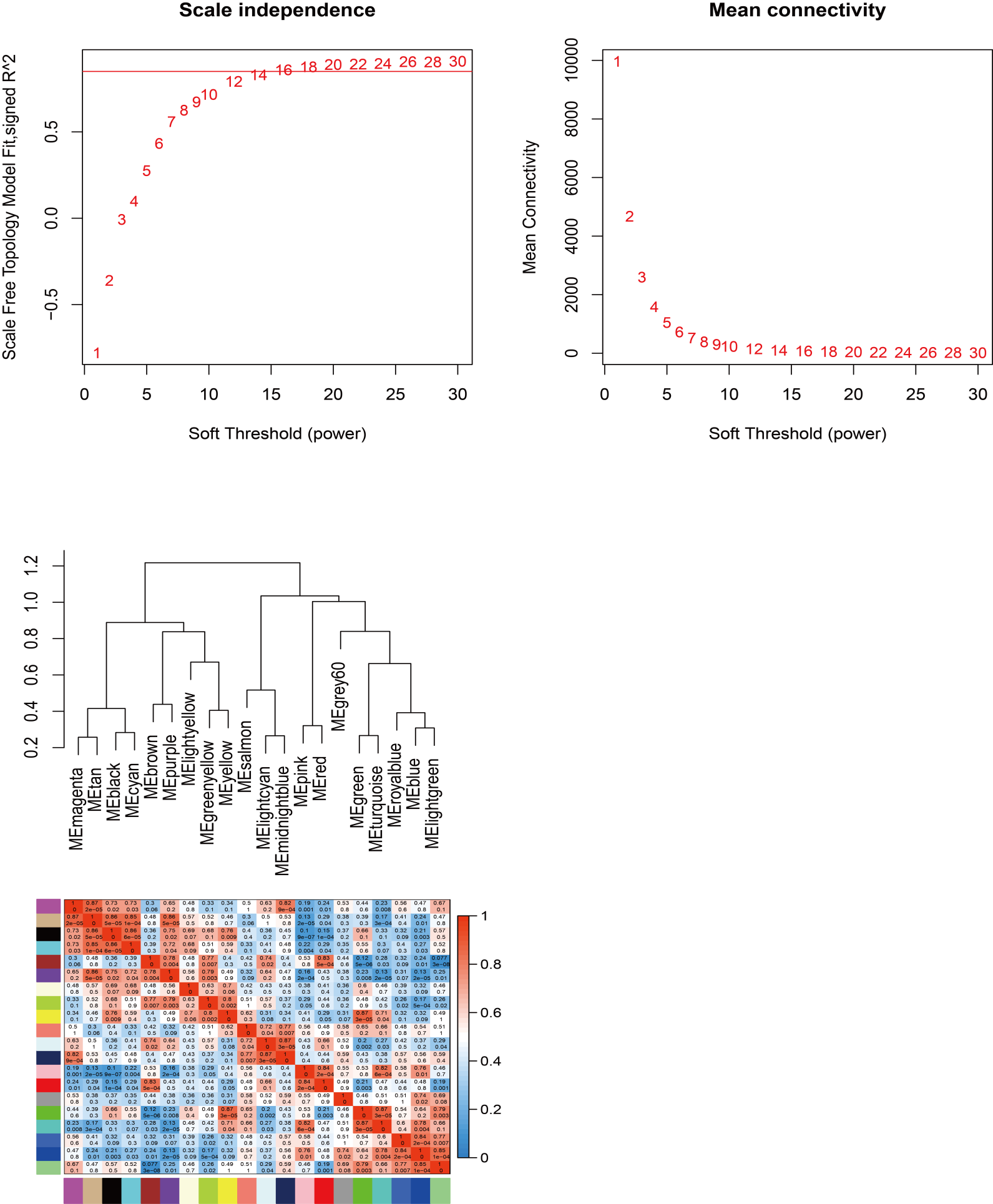


**Fig.S7.**

**c**

**a**

**b**

433466666

**Fig.S8.**


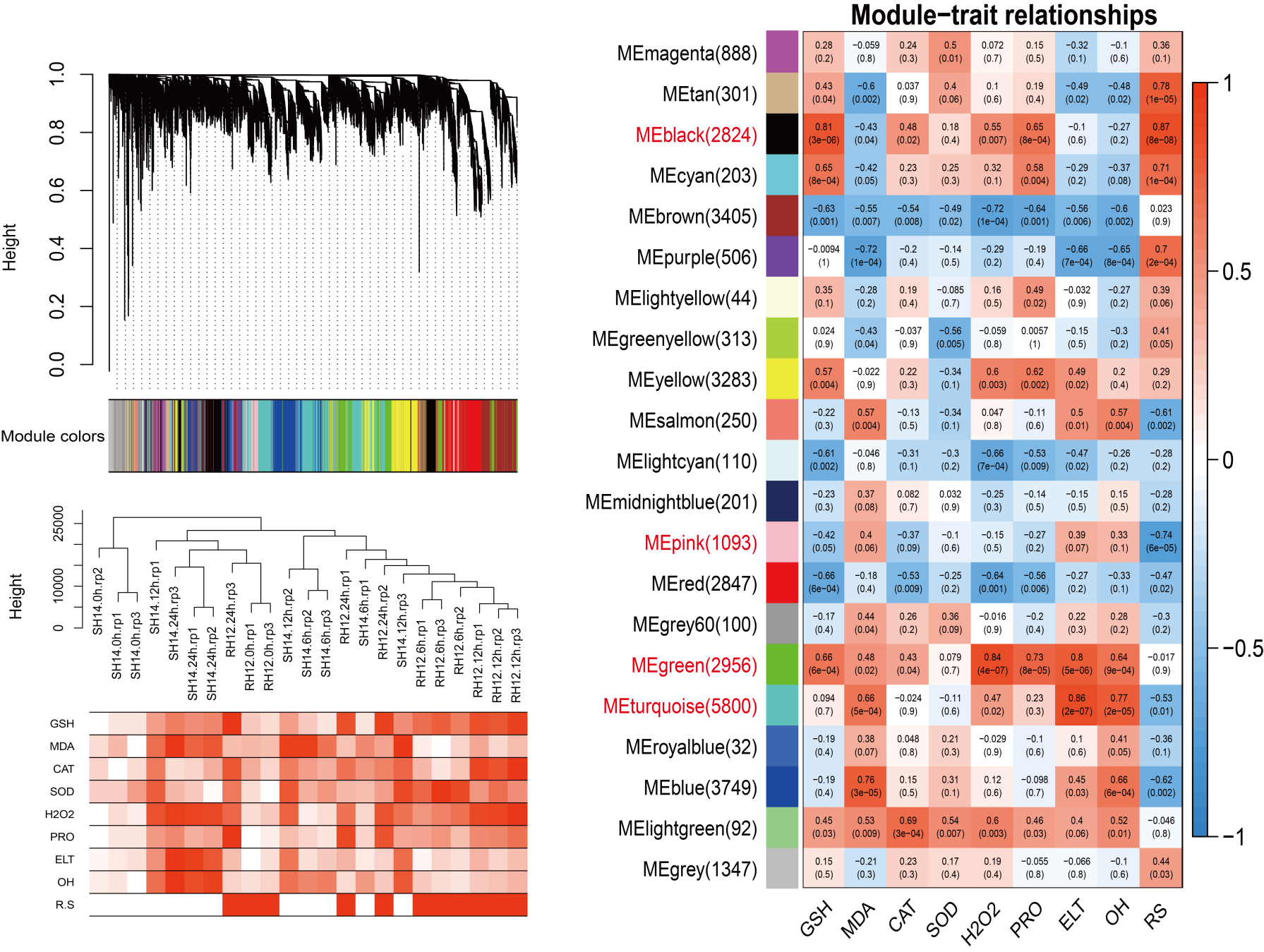


**a**

**b**

**c**

**Fig.S9.**


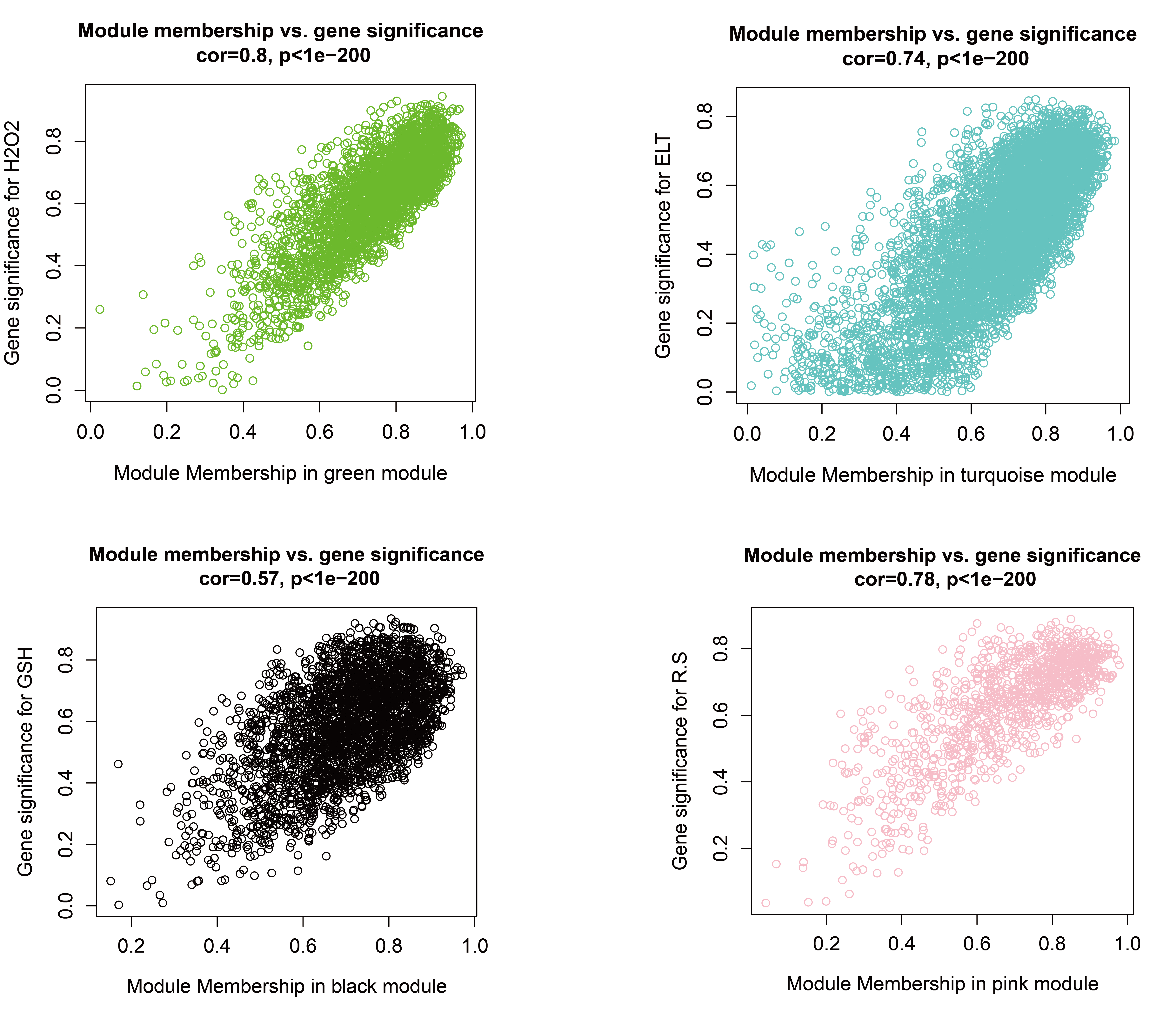

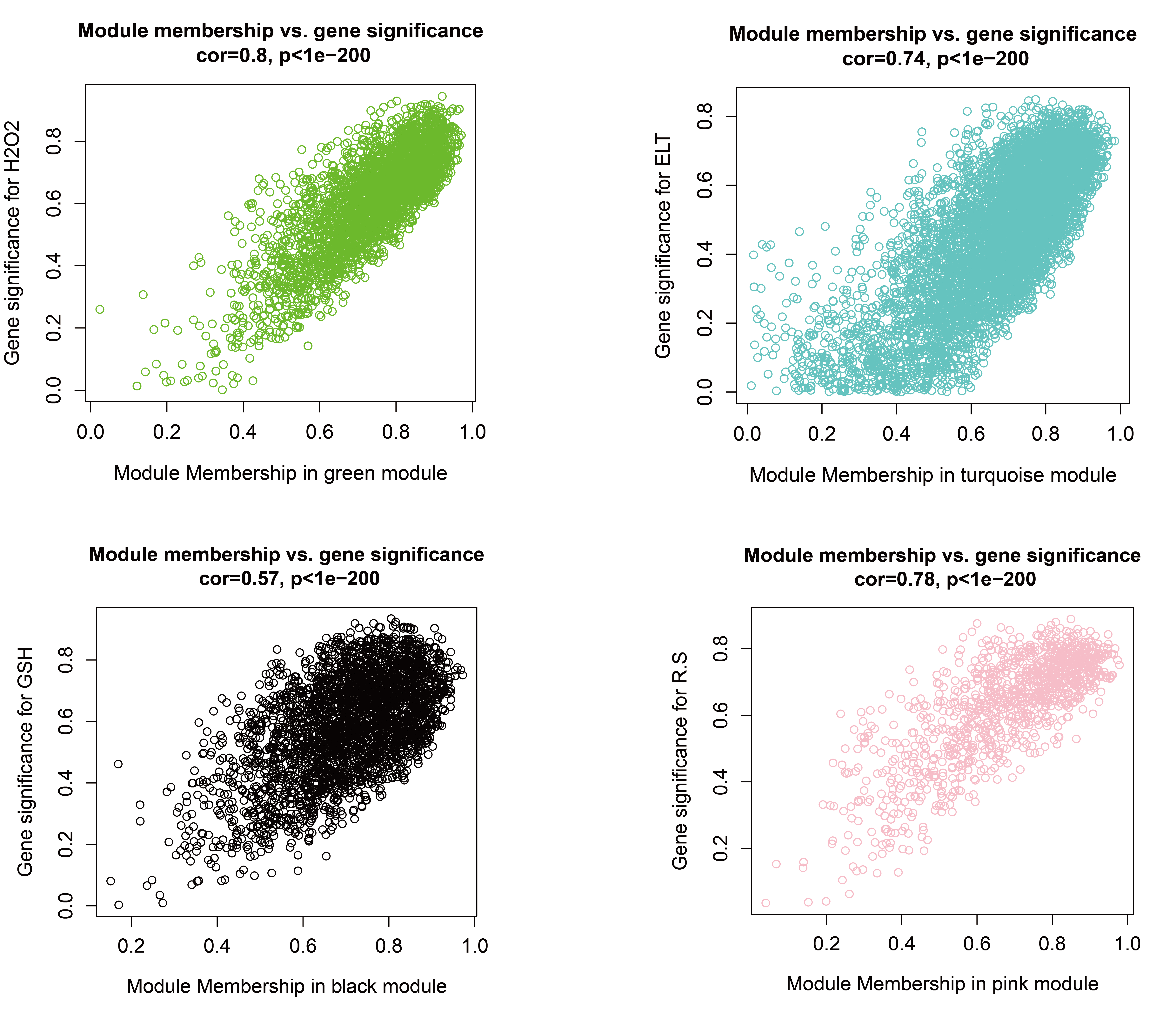


**a**

**d**

**c**

**b**


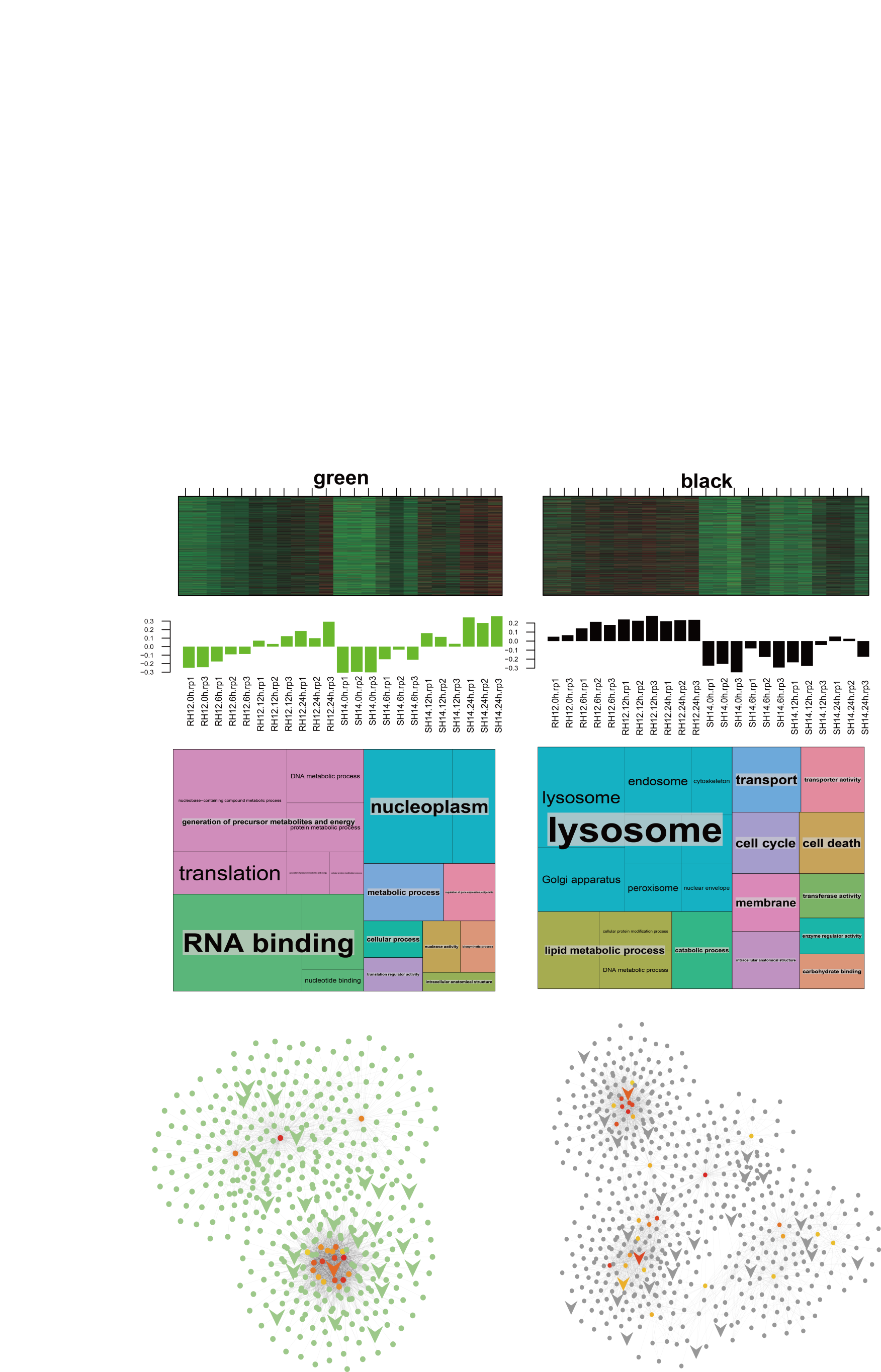


**a**

**e**

**d**

**c**

**f**

**b**

***AcREM14***

***AcZML1***

***AcSPT***

***AcC3H1***

**Fig.S10.**


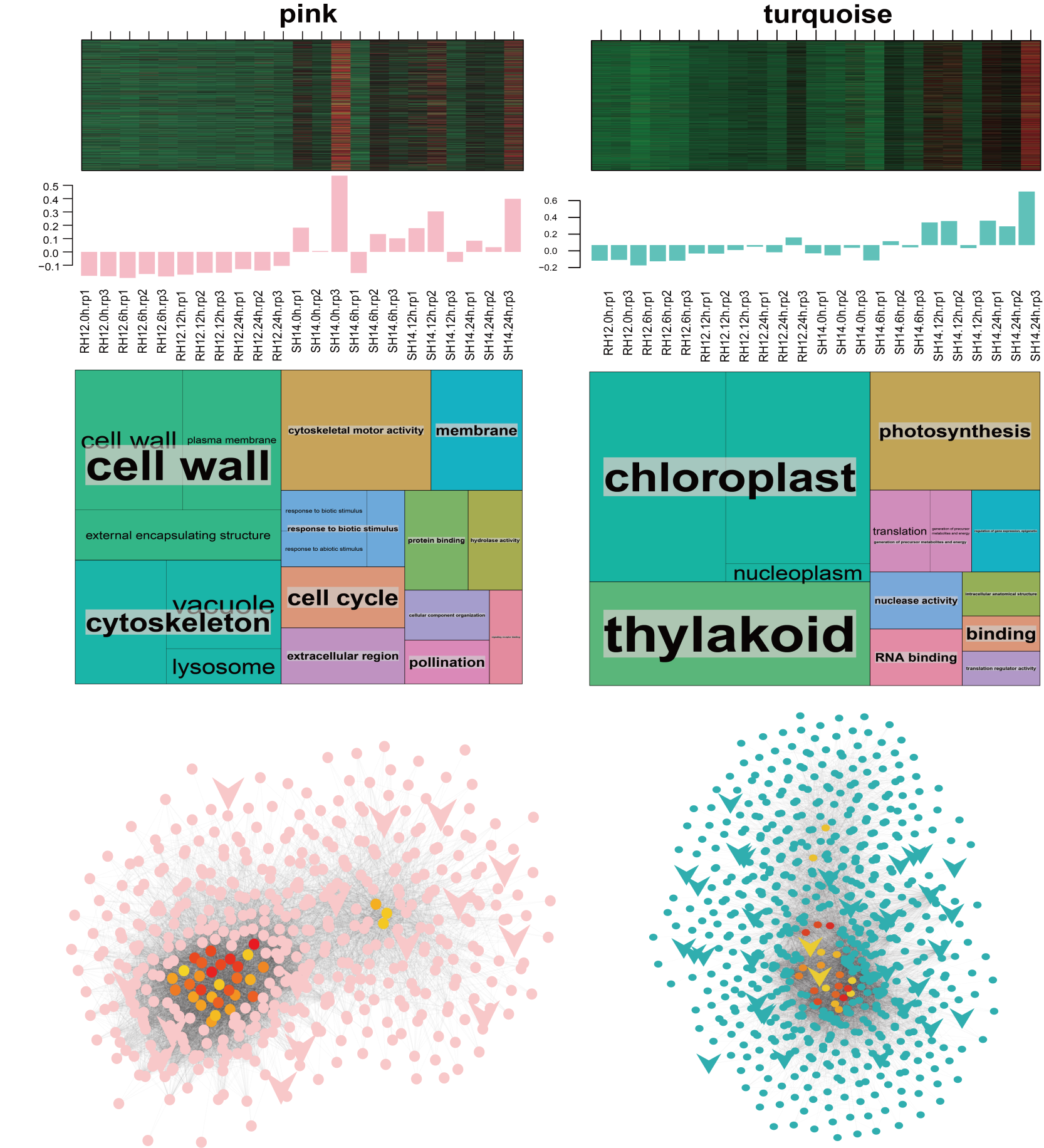


**a**

**b**

**f**

**c**

**d**

**e**

***AcAS1***

***AcZFP1***

**Fig.S11.**


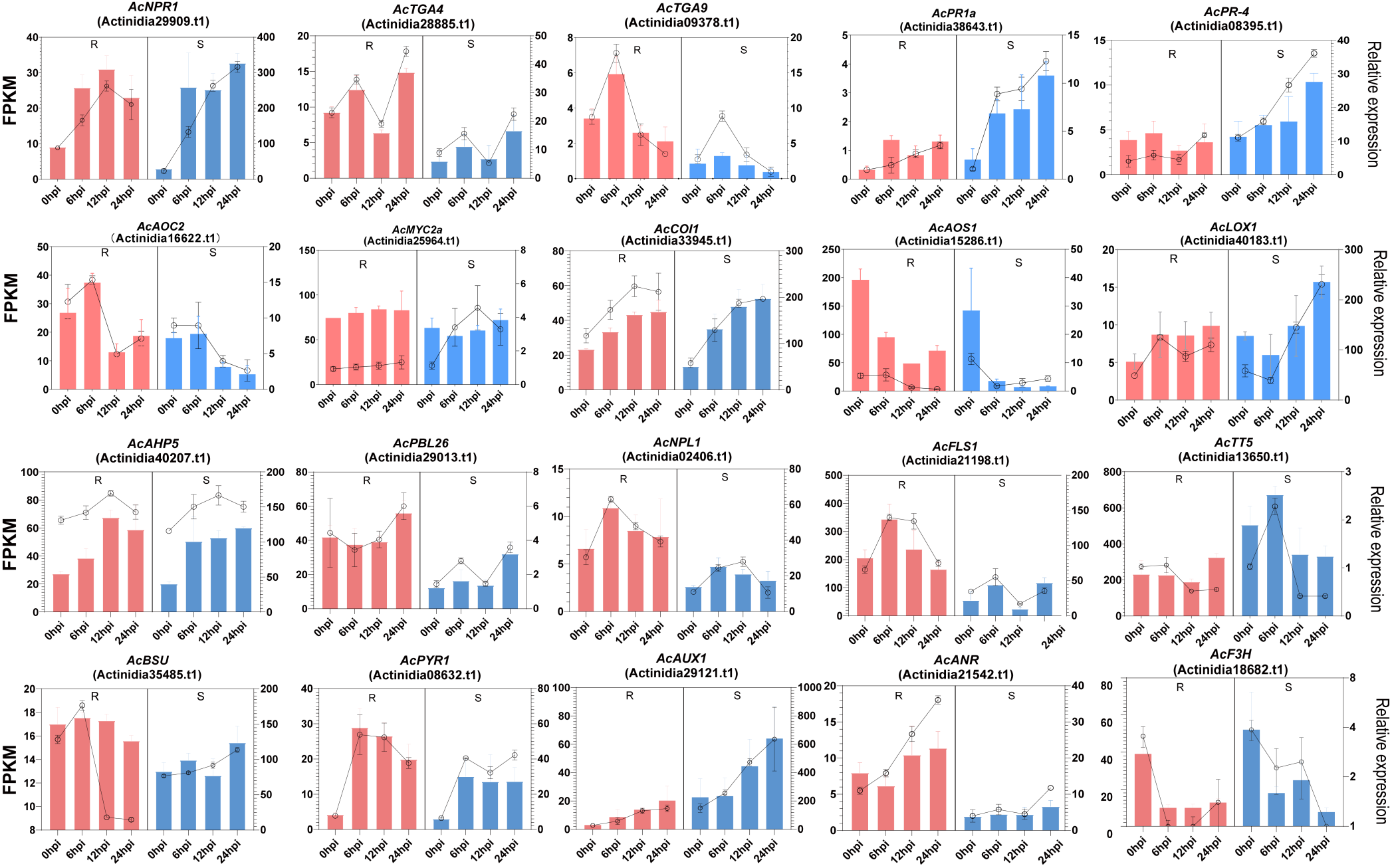


**Fig.S12.**


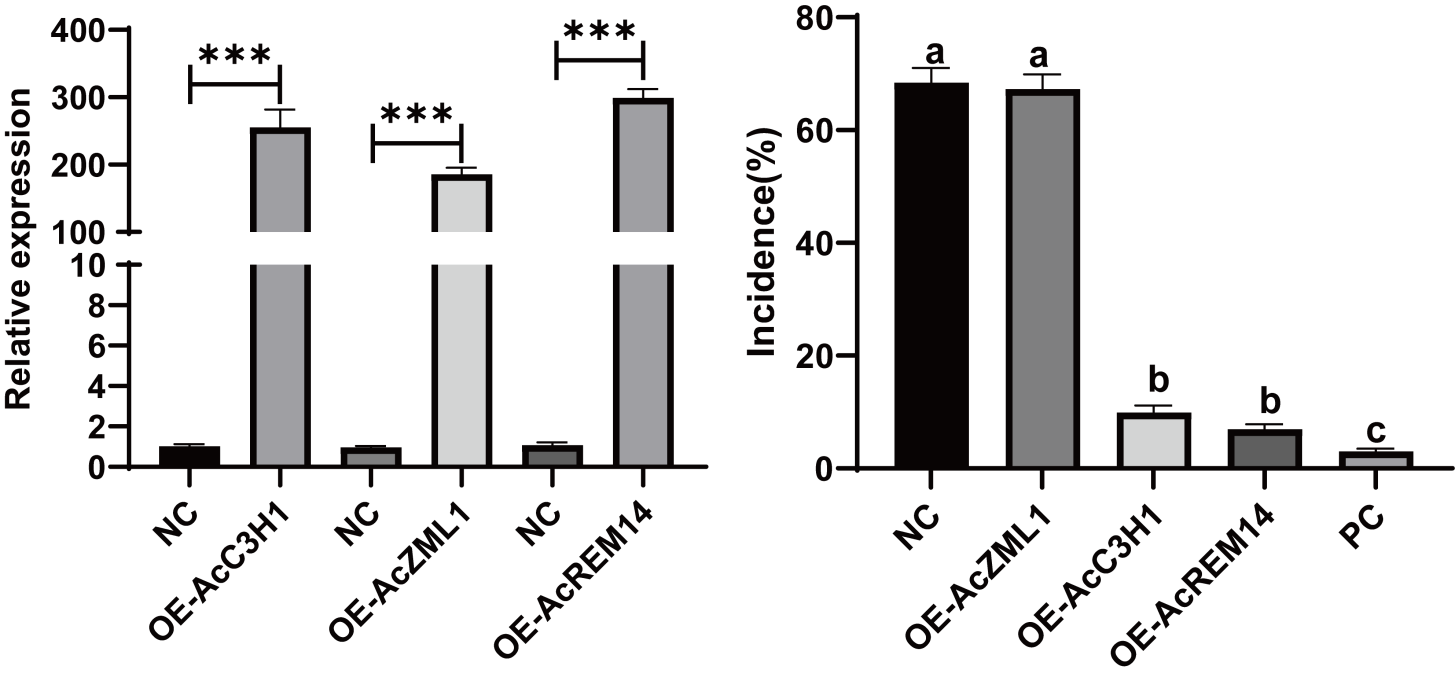


**a**

**b**

**Fig.S13.**

**Fig.S14.**


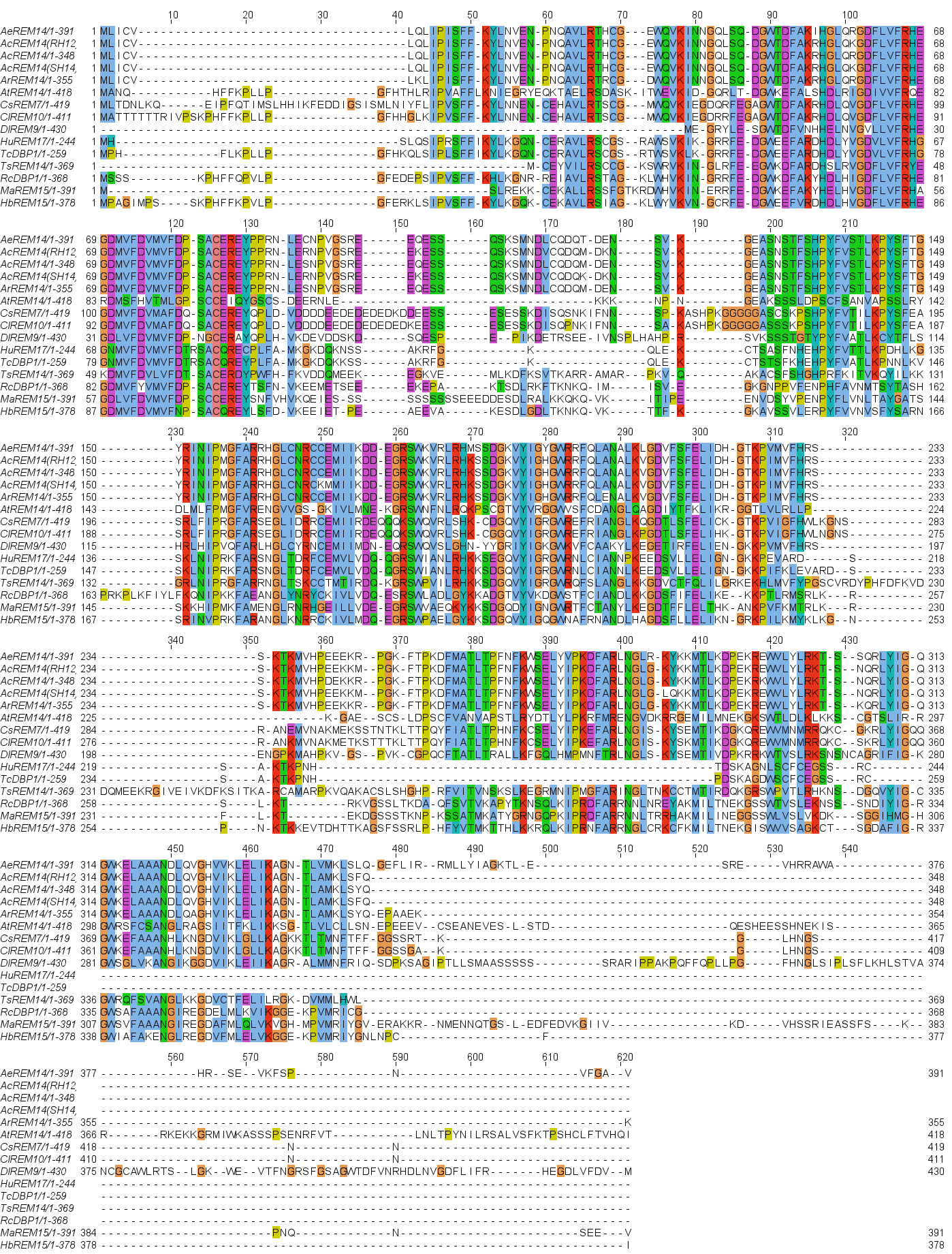


**Kiwifruit**

**Kiwifruit**


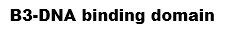

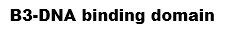

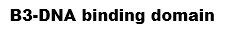


**Other species**

**Other species**

**Other species**

**Other species**

**Kiwifruit**


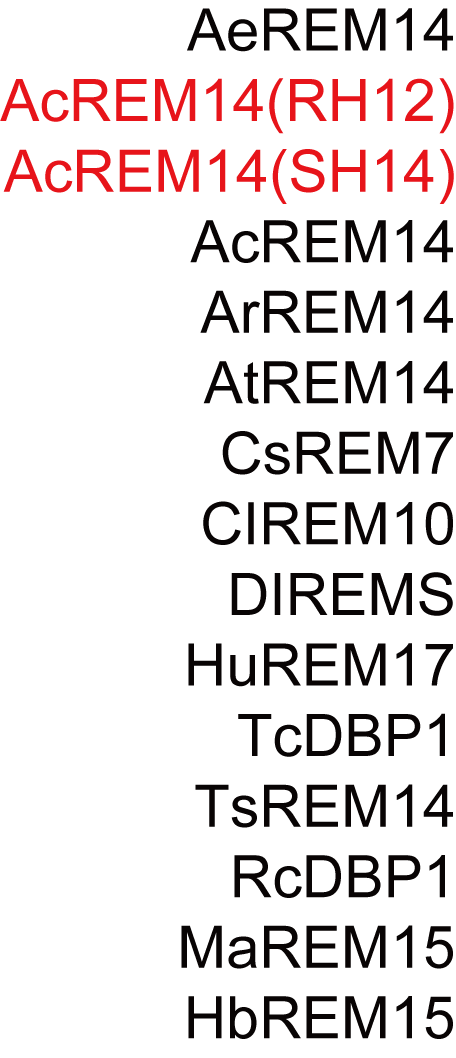

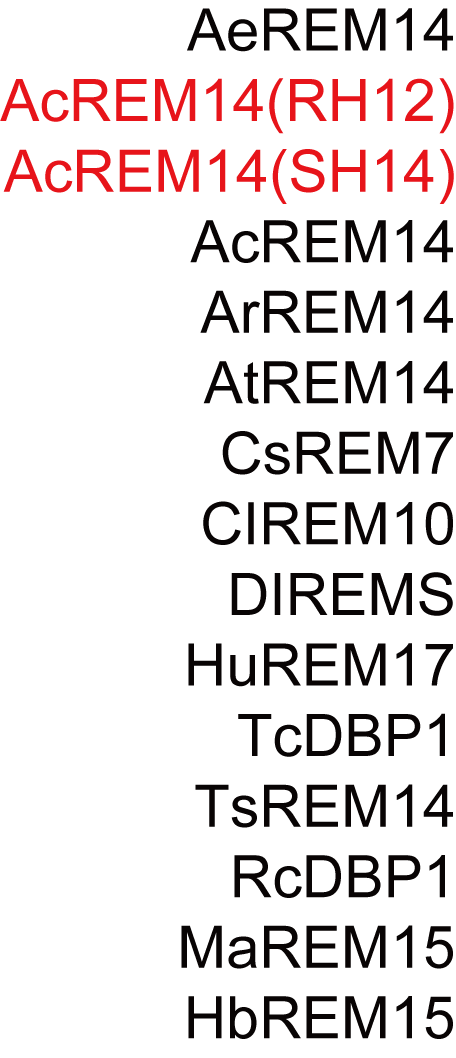

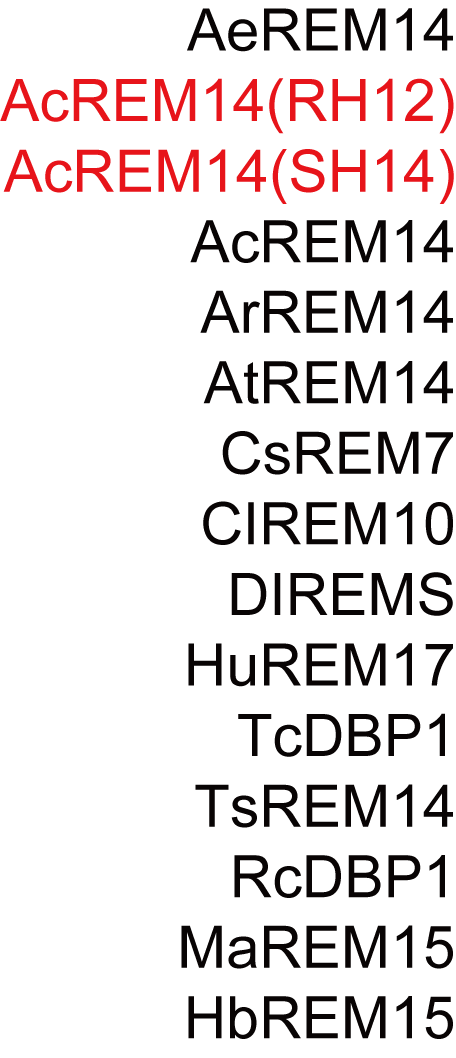

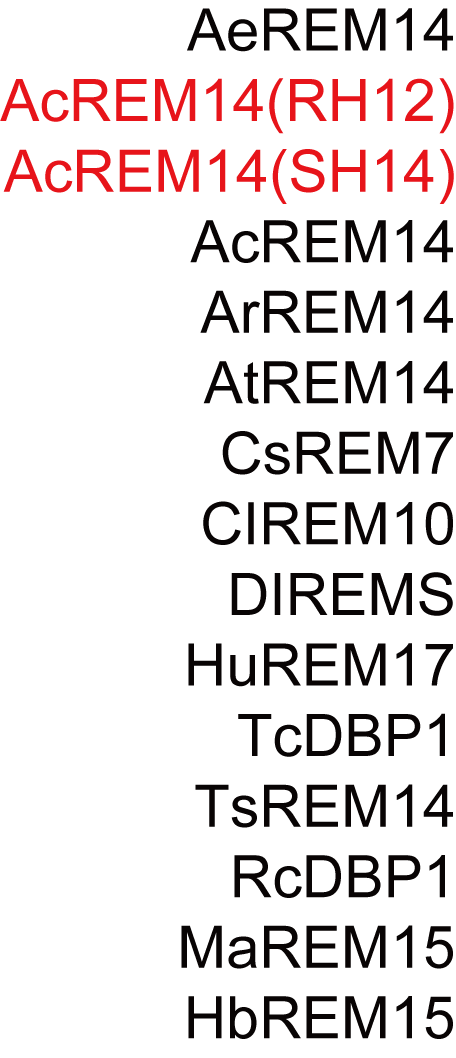

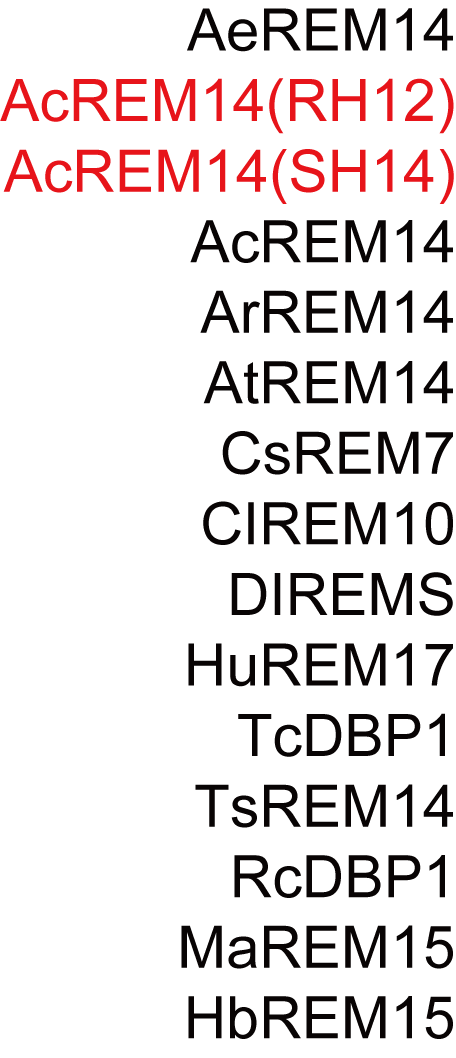


**Other species**

**Kiwifruit**

**Kiwifruit**

**Other species**

**Kiwifruit**


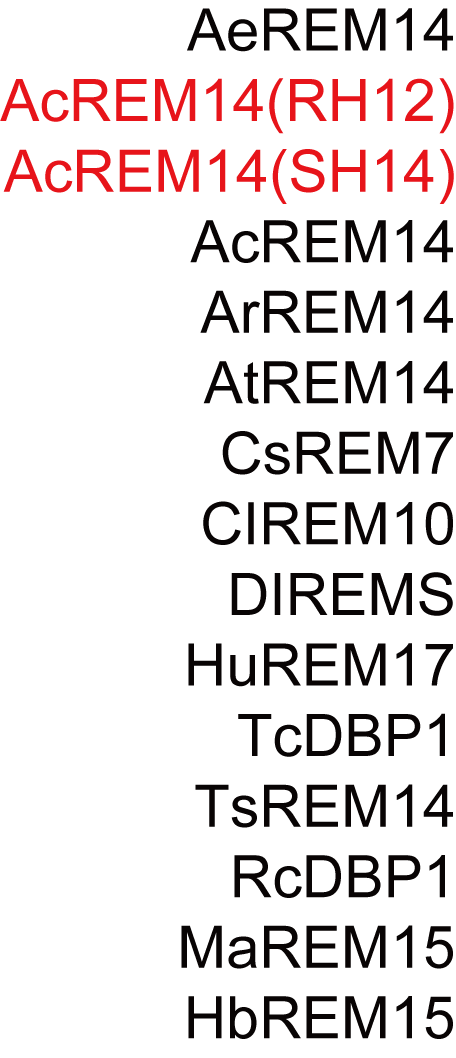


**Fig.S15.**


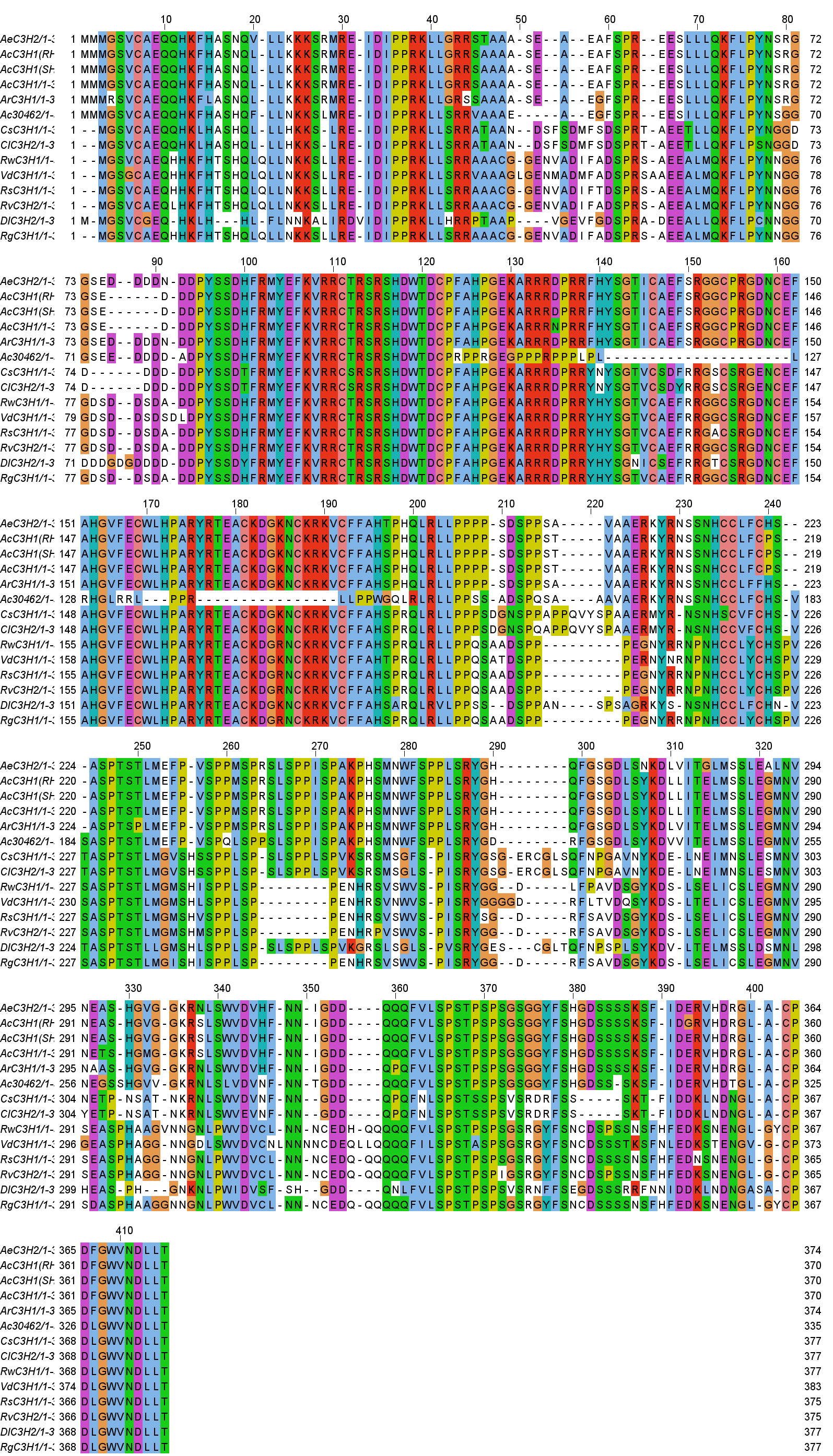


**Kiwifruit**

**Other species**


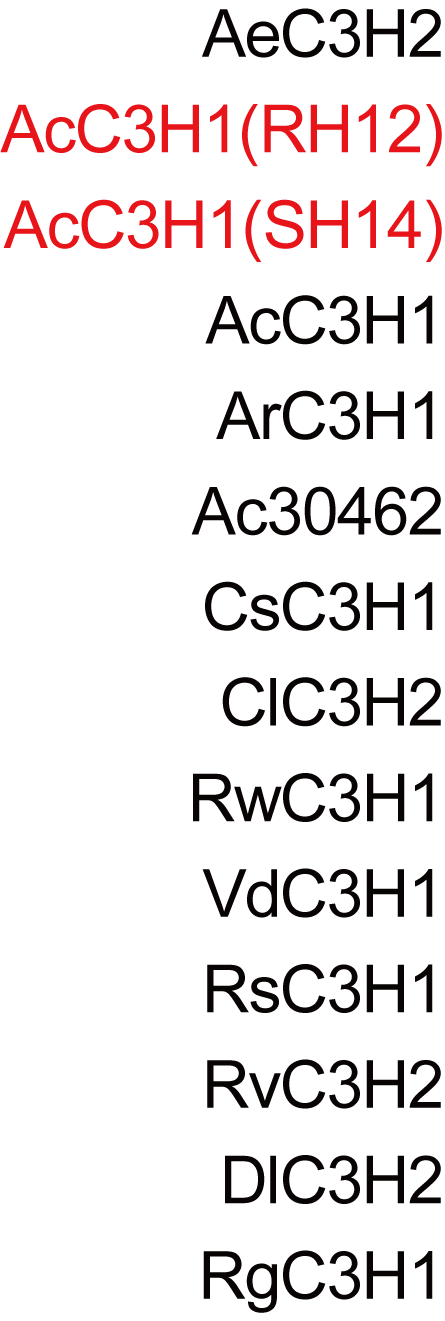

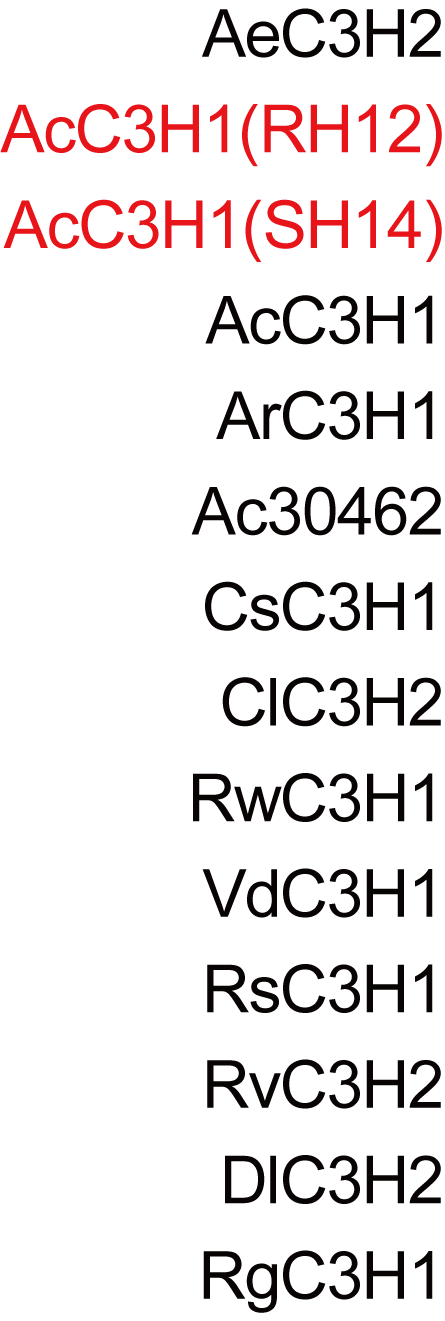

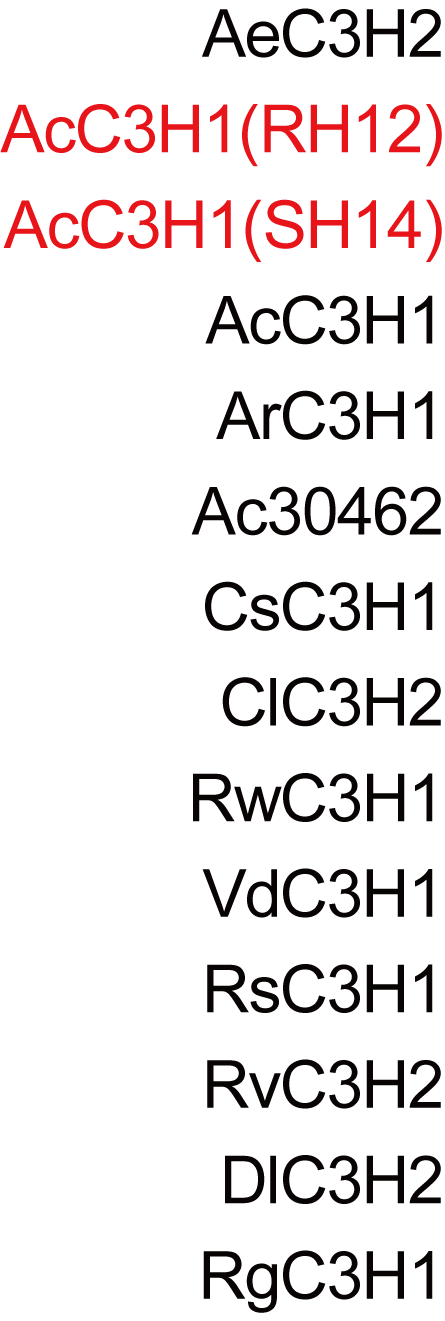

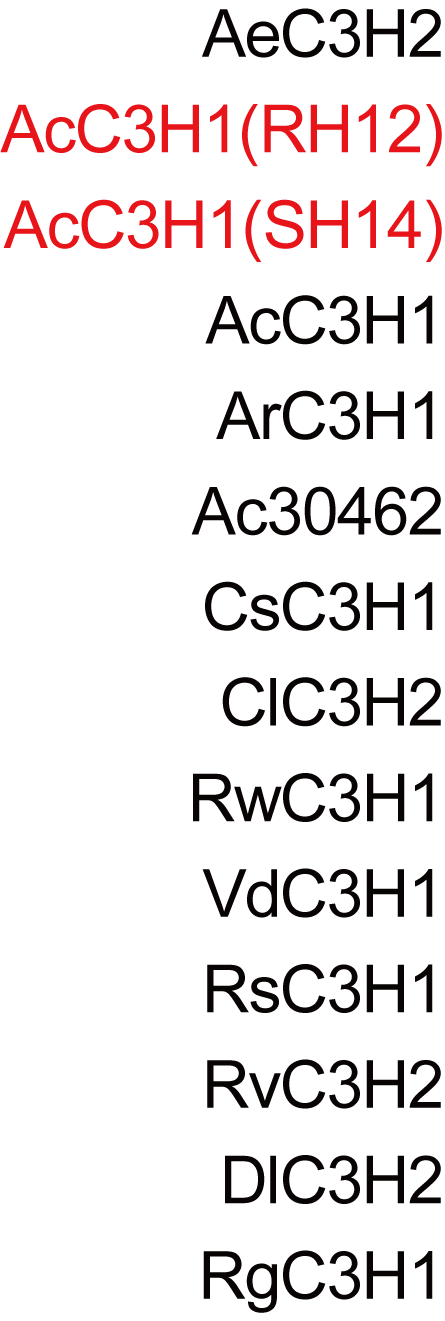

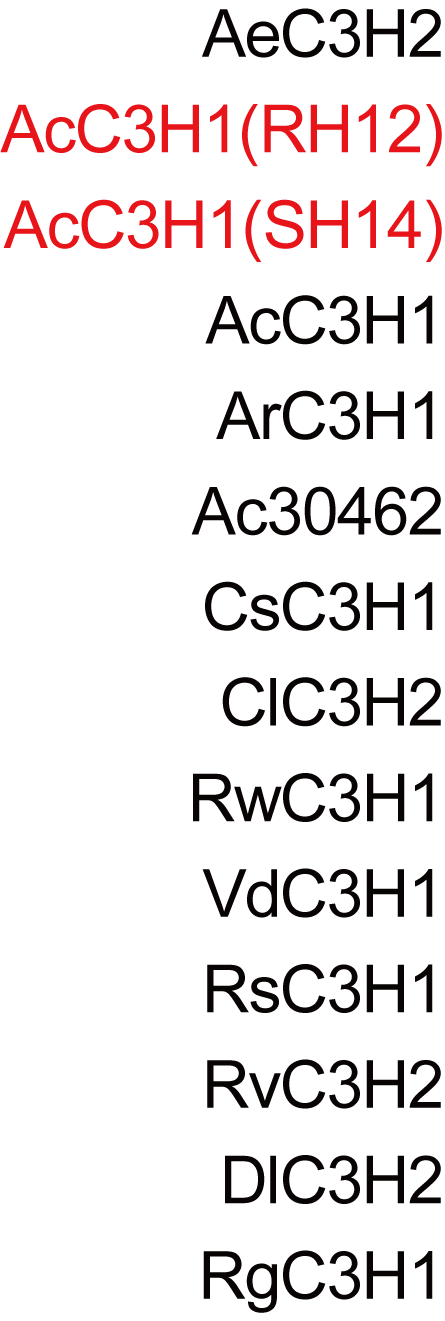

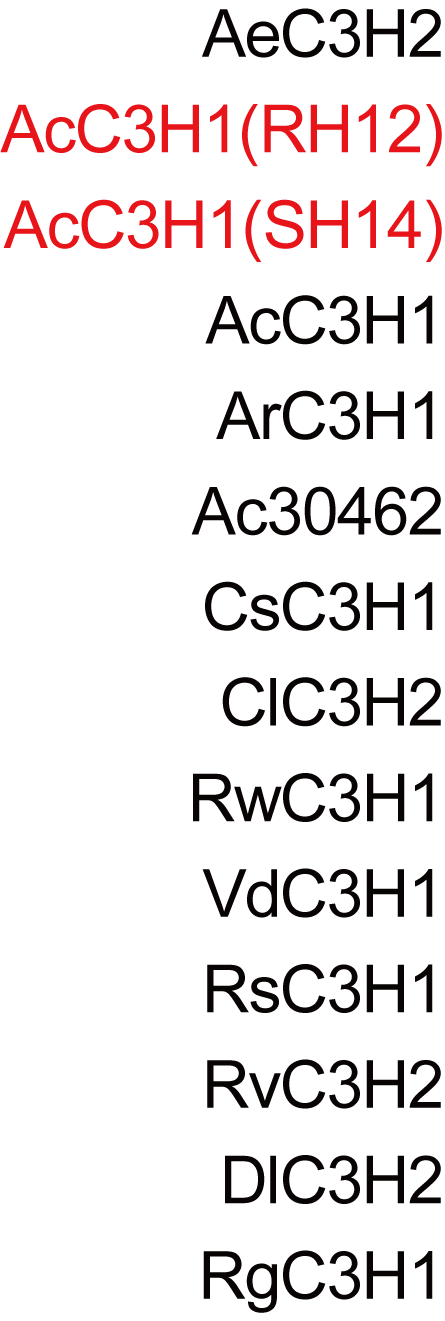


**Kiwifruit**

**Other species**

**Kiwifruit**

**Other species**

**Kiwifruit**

**Other species**

**Kiwifruit**

**Other species**

**Kiwifruit**

**Other species**


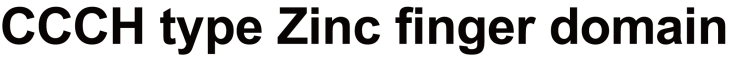


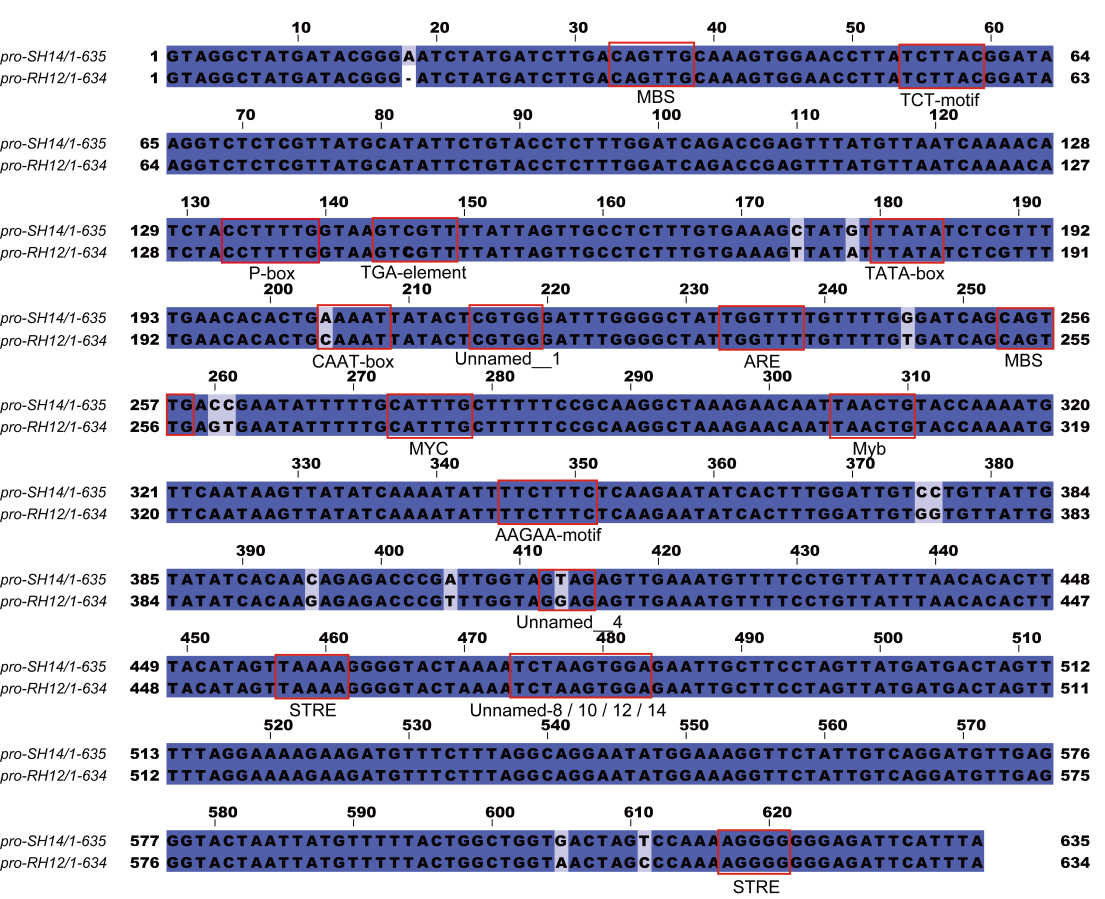

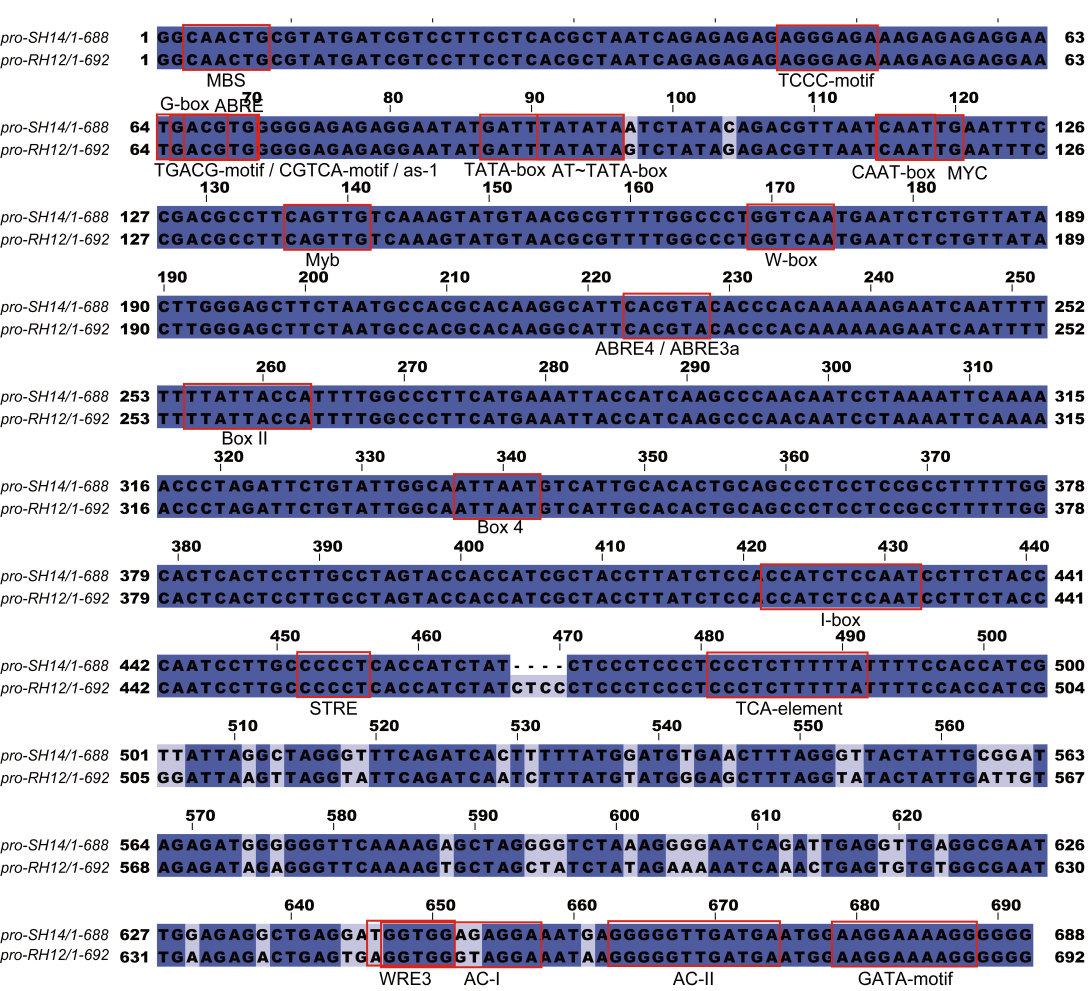


**ATG**

**ATG**

**a**

**b**

**b**

**Fig.S16.**

a
